# Performance Analysis of Discrete Wavelet Transform Bases for Multimodal Medical Image Decomposition and Fusion Quality Assessment

**DOI:** 10.3390/jimaging12080340

**Published:** 2026-07-27

**Authors:** Stojche Rechanoski, Jasmina Veta Buralieva, Saso Koceski, Nikolay Hinov

**Affiliations:** 1 Faculty of Computer Science, Goce Delcev University, Krste Misirkov, 10A, 2000 Stip, North Macedonia; stojce.31027@student.ugd.edu.mk (S.R.); jasmina.buralieva@ugd.edu.mk (J.V.B.); 2 CoE “National Center of Mechatronics and Clean Technologies”, 1000 Sofia, Bulgaria; 3 Faculty of Computer Systems and Technologies, Technical University of Sofia, 1756 Sofia, Bulgaria

**Keywords:** wavelet transform, medical images, wavelet-based image decomposition and fusion

## Abstract

The fusion of information from multiple imaging modalities plays a very important role in medical diagnosis. Wavelet-based transformations have been identified as powerful methods for this purpose due to their multiresolution nature, which enables the simultaneous preservation of both structural and fine-detail information across different frequency bands. In this paper we present experimental results obtained from the wavelet-based decomposition and fusion of medical images using Python and PyWavelets. Seven wavelets from four wavelet families—Daubechies (‘db1’, ‘db10’), biorthogonal (‘bior1.3’, ‘bior4.4’), coiflets (‘coif1’, ‘coif10’), and discrete Meyer (‘dmey’)—were systematically evaluated across three decomposition levels. An emphasis was put on the preservation of approximation and detail sub-images. Results outline that simpler wavelets used for the wavelet-based decomposition of grayscale medical images produce more details when compared with the colored medical images. From the tested fusion rules, and for the specific image pairs used in the analyses, we conclude that the average fusion rule gives the best information, without a lack of or excess of information regarding the visual quality of the fused image. Considering entropy as a quality metric and according to its higher values at all levels, the ‘bior4.4’ wavelet emerges as the best for wavelet-based image fusion. These findings could provide practical guidance for wavelet selection in multimodal medical image fusion pipelines.

## 1. Introduction

Modern medicine deals heavily with medical images for diagnosis and decision-making, which are typically analyzed digitally. Image processing techniques applied to raw medical images can reveal valuable information and support medical professionals [1]. As demonstrated by numerous studies, wavelets have become a powerful and efficient mathematical tool in medical image processing, with applications such as image compression, denoising, fusion, and segmentation. A review of the applications of the wavelet transform in digital image processing can be found in [2].

Due to the large volume of medical image data, wavelet-based methods have been widely investigated for image compression, particularly for magnetic resonance imagery (MRI) and computed tomography (CT) images, often outperforming conventional codecs such as JPEG [3,4,5]. For example, an improved wavelet-difference-reduction-based medical image compression algorithm achieves lossless compression of color histopathological microscopy images [6]. In addition, multiresolution wavelet analysis has proven effective for medical image denoising, improving image quality metrics such as peak signal-to-noise ratio (PSNR) and signal-to-noise ratio (SNR) in ultrasound and MRI data [7,8]. A very effective approach to image denoising emerges from pairing the wavelet transform with a convolutional neural network (CNN) [9]. Another hybrid method combines the discrete wavelet transform (DWT) with Gaussian filtering for image denoising [10].

The DWT is also used to improve the efficiency of medical image translation and generation. A great example is a state-of-the-art framework [11] that leverages wavelet-based diffusion for cross-modality medical image generation.

In image segmentation, multiresolution wavelet analysis has demonstrated effectiveness, with extensions such as ridglet and curvelet transforms addressing limitations in capturing directional and edge features in CT images [12]. Additionally, related approaches have explored image enhancement and coloring of grayscale medical images using filter-based techniques [13]. Wavelet-based feature extraction has been shown to significantly influence recognition performance, with both the choice of wavelet basis and decomposition level playing a critical role in classification accuracy [14].

DWT has also been used as a part of a state-of-the-art medical image classification system that also incorporates group-convolutional neural networks (G-CNNs) [15]. A recent review on the applications of the wavelet transform in radiomic feature extraction from CT, MRI, and positron emission tomography (PET) images [16] emphasizes the importance of the DWT and its more advanced variants for quality improvements in radiomic analyses. The same study also confirms that MATLAB and Python are the most popular computational tools among researchers in the field.

Wavelet transforms have also been widely applied to medical image fusion, enabling the integration of complementary information from different imaging modalities such as PET and MRI [17,18,19,20]. A wavelet transform-based system for brain tumor detection from fused CT and MRI images is presented in [21]. The authors of a study [22] evaluate CNN-based fusion of medical images using the DWT as one of the conventional methods against which the CNN approach is compared. In another study [23], the performance of a novel discrete component wavelet transform is compared with conventional fusion techniques, also including the DWT. An analysis of the DWT variants is presented in a study [24], where the authors conclude that the Daubechies ‘db1’, Reverse Biorthogonal ‘rbio1.1’, and ‘Haar’ wavelets provide the best visual quality for the fusion result. The DWT has been used in medical image fusion in a variety of forms and with different fusion criteria [25]. A multimodal medical image fusion scheme based on an optimized dual-tree complex wavelet transform (ODTCWT) is presented in [26], where the source images are decomposed into low- and high-frequency coefficients and a hybrid optimizer is used for tuning the transform’s filter coefficients. The high-frequency coefficients are fused using an adaptive weighted-average rule with optimized weights, while the low-frequency coefficients are fused with the average fusion rule. The fused image is reconstructed via the inverse transform, with the objective of maximizing the fused mutual information. The fusion quality metrics are important as different fusion algorithms inherently introduce limitations. An example of a proposed perceptual no-reference multi-scale-aware attention network for quality evaluation of fused medical images can be found in [27]. A novel approach to 3D multi-modal medical image fusion based on a 3D multiscale wavelet convolutional neural network, outperforming state-of-the-art models in terms of subjective and objective fusion quality, can be found in [28]. Another novel approach to medical image fusion, combining a wavelet transform and a deep convolutional neural network, can be found in [29]. An example of recent research to assess the performance of various fusion rules can be seen in a study [30] that provides an analysis of classical and deep learning-based fusion strategies, while also considering both symmetric and asymmetric fusion. An interesting paper [31] presents an FPGA hardware-based platform for medical image fusion leveraging relative total variation decomposition, while comparing the results with traditional techniques among which the DWT is also mentioned. The development of new fusion algorithms is always emerging as a necessity. A recent study [32] proposes the hybrid use of multi-scale cosine similarity decomposition (MCSD) and the DWT, leading to improvements in the evaluated fusion quality metrics. Image fusion finds many applications. It is worth mentioning that image fusion supported by Laplacian and Gaussian pyramid decomposition and reconstruction can effectively enhance the quality of low-light images [33]. A convolutional neural network-based approach for improving overall quality in low-light images has also been proposed [34].

DWT-based steganography has also been used as part of a framework to improve security in medical internet of things (IoT) environments [35]. Evidently, there are many examples in existing recent literature where wavelets are used as part of innovative solutions with a variety of applications to medical image processing. However, the selection of the most suitable wavelet basis always persists as a challenge and a research gap [36], which can surely be interpreted as motivation for further studies on the topic.

In the present paper, we use the availability of high-level programming environments such as Python to analyze medical images. Motivated by the broad applicability of wavelets, the study investigates selected functionalities of the PyWavelets library, focusing on medical image decomposition and fusion, while presenting a comparative analysis of results obtained when using different wavelet families and decomposition levels.

To the best of our knowledge, no prior study has investigated the potential of Python and PyWavelets as an aid in the assessment of the influence of the wavelet family selection and the fusion rule choice on the quality of the fused brain medical image across multiple decomposition levels. Further, the question of whether higher-order wavelets with compact support and symmetry properties, such as biorthogonal wavelets, perform better than simpler wavelets in fusion, such as the Haar or lower-order Daubechies wavelets, is still somewhat unclear. Therefore, the present study, motivated by these gaps, aims to provide comparative evidence that could potentially benefit more complex wavelet-based medical image processing pipelines, while also exploring the research potential of Python and PyWavelets in this field. The fact that this study employs Python and PyWavelets, which are open-source and accessible to a wide range of researchers, effectively positions this study as a catalyst for further research and improvements on the topic.

## 2. Preliminaries

### 2.1. Wavelet and Wavelet Transform

The term wavelet refers to a small wave that can be used to apply transformations to a signal in order to achieve time–frequency analysis [37]. A function ψ is called a wavelet if it satisfies $\int_{-\infty }^{+\infty }\psi \left(x\right)dx=0.\;$ The wavelet transform is defined with respect to the following family of wavelets $\psi_{a,b}\left(t\right)=\frac{1}{\sqrt{\left|a\right|}}\psi \left(\frac{t-b}{a}\right),\;a,b\in R,a\neq 0$, where ψ is a wavelet, a is known as the scaling parameter, and b is known as the translation parameter, corresponding to the compression or scale and time location of the wavelet, respectively. A wavelet ψ(t) has n vanishing moments if $\int_{-\infty }^{\infty }t^{k}\psi \left(t\right)dt=0,\;$ for 0<k<n, [38].

The wavelet transforms can mainly be divided into two categories: continuous and discrete. The continuous wavelet transform imposes more difficulties in its application when compared with the discrete wavelet transform (DWT), hence, the DWT is used more often. However, it should be noted that the signal itself is still continuous [39]. The DWT is achieved after applying the changes for the scaling and translation parameters in discrete steps [37]. The continuous transform of a square integrable function f is defined as ${\;W}_{\psi }f\left(b,a\right)=\int_{-\infty }^{+\infty }f\left(x\right)\bar{\psi_{a,b}}\left(x\right)dx,\;\;a,b\in R,a\neq 0,$ where $\bar{\psi_{a,b}}$ is the complex conjugate function, while the discrete wavelet transform is defined as $\sum_{j\in Z}\sum_{k\in Z}d_{j,k}\psi_{j,k}j,k\in Z,$ such that $d_{j,k}=\int_{-\infty }^{+\infty }f\left(x\right)\bar{\psi_{j,k}}\left(x\right)dx,\;$ are the wavelet coefficients, and $\psi_{j,k}\left(x\right)=\;2^{j/2}\psi \left(2^{j}x-k\right)$. An overview of the basic concepts behind the wavelet transforms can be found in [38,40].

### 2.2. Decomposition and Fusion

**Figure 1:**
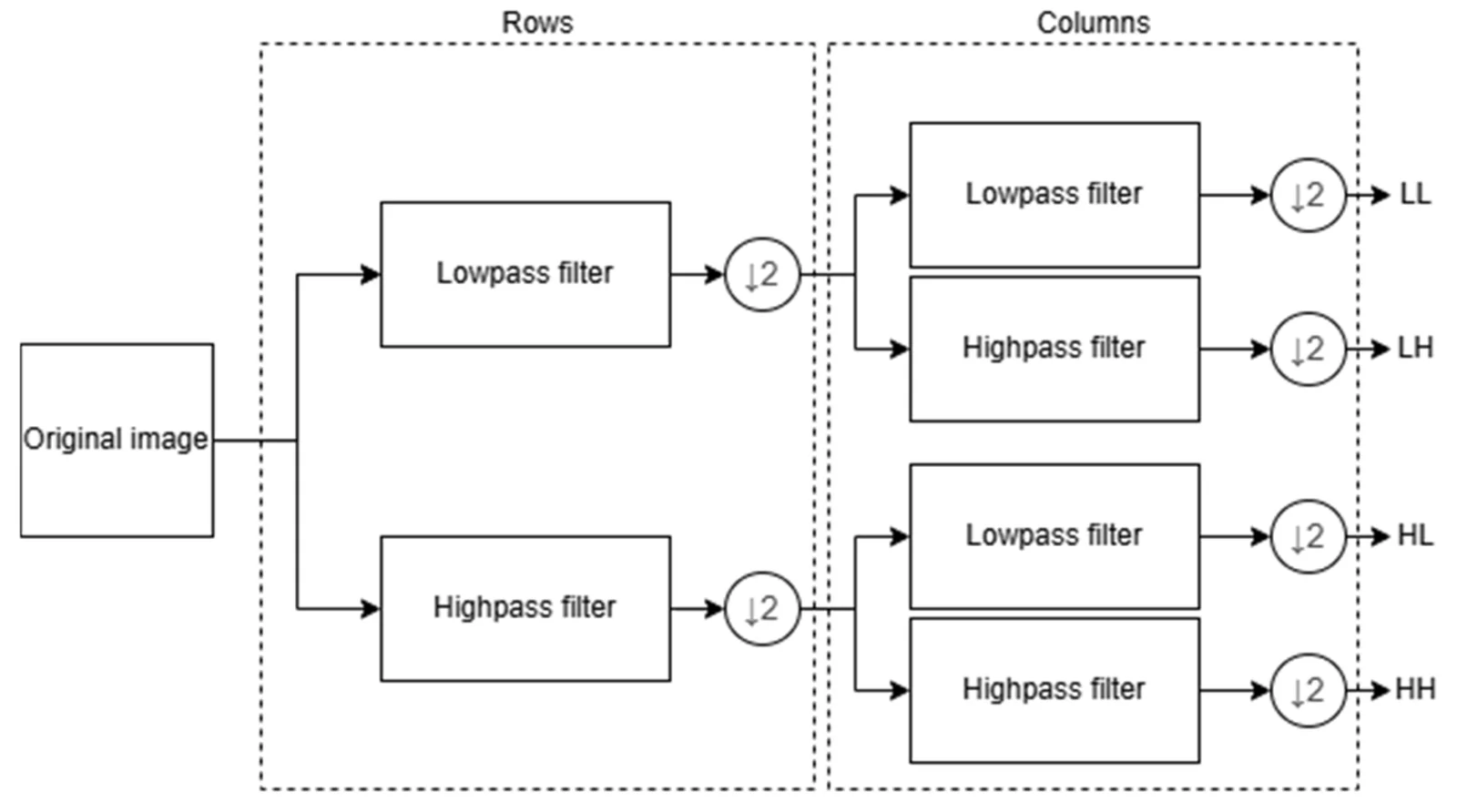
One-level image decomposition, adapted from [31,33]. The horizontal arrows indicate the flow in the diagram, the vertical downward arrows indicate downsampling by a factor of 2.

A common representation of the DWT is expressed through filter banks. Namely, four new sub-images are produced out of the original image. As illustrated in Figure 1, the approximation (LL) image is formed after lowpass filtering is applied to the rows and the columns of the original image. The approximation image represents the low-frequency coefficients, while the other sub-images represent the high-frequency or detail coefficients, i.e., LH (horizontal features), HL (vertical features), and HH (diagonal features). This approximation image would be the input for the next level of decomposition [24,39].

After the coefficients have been computed through DWT for different images, image fusion based on a fusion rule can be performed. Some of the basic image fusion rules are the average, minimum, and maximum fusion rules. If the average rule is used, the fused image is obtained by taking the average of the respective pixels from the corresponding to-be-fused images. If the minimum fusion rule is used, then the pixel with the lowest intensity value will be chosen from both images; on the contrary, if the maximum fusion rule is used, then the pixel with the highest intensity value will be selected from both images (see example [37]). Then, after the fused coefficients have been obtained, the fused image can be constructed by using the inverse discrete wavelet transform (IDWT) [24].

The quality of the fused image can be assessed through both qualitative or subjective, and quantitative or objective criteria. Quantitative evaluation can be performed with or without a reference image. In the absence of a reference image, some of the useful metrics for quality assessment of the fused image are entropy, standard deviation, and mutual information (MI). Entropy measures the quantity of information contained in an image. Higher entropy values may indicate better fusion as the fused image contains more information. Standard deviation (Std Dev) measures the intensity variation (i.e., contrast) in the fused image. Higher values are better, as they represent higher contrast. Mutual information (MI) represents the similarity of two images. Higher values of MI denote better fusion [41,42].

## 3. Materials and Methods

### 3.1. Data

**Figure 2:**
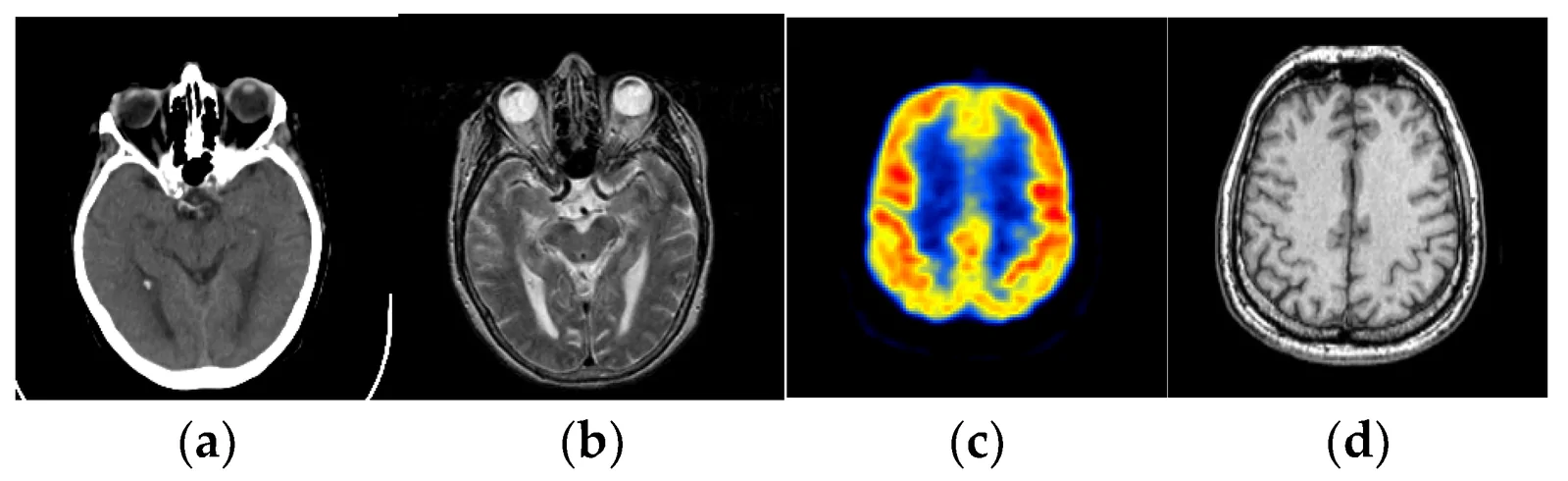
The original image for (**a**) CT; (**b**) MRI; (**c**) PET; (**d**) MRI, where (**a**), (**b**), and (**d**) are grayscale intensity images where pixel brightness indicates the X-ray attenuation (CT) and MR signal intensity (MRI), while (**c**) is a PET image in which warm hues indicate high and cool hues indicate low radiotracer uptake.

In this paper, we provide the results for wavelet-based image decomposition and fusion using the PyWavelets module. The original images for brain CT, MRI, PET, and a second MRI image with a different cross-section of the brain, are given in Figure 2a,b,c, and d respectively. The images are taken from a publicly available dataset, available from a GitHub repository [43] that includes paired brain images of the mentioned modalities, making it convenient for research in image fusion. The size of the CT, MRI, and PET images from the dataset is 256 × 256 pixels.

### 3.2. Grayscale and YUV Color Space Conversion

**Figure 3:**
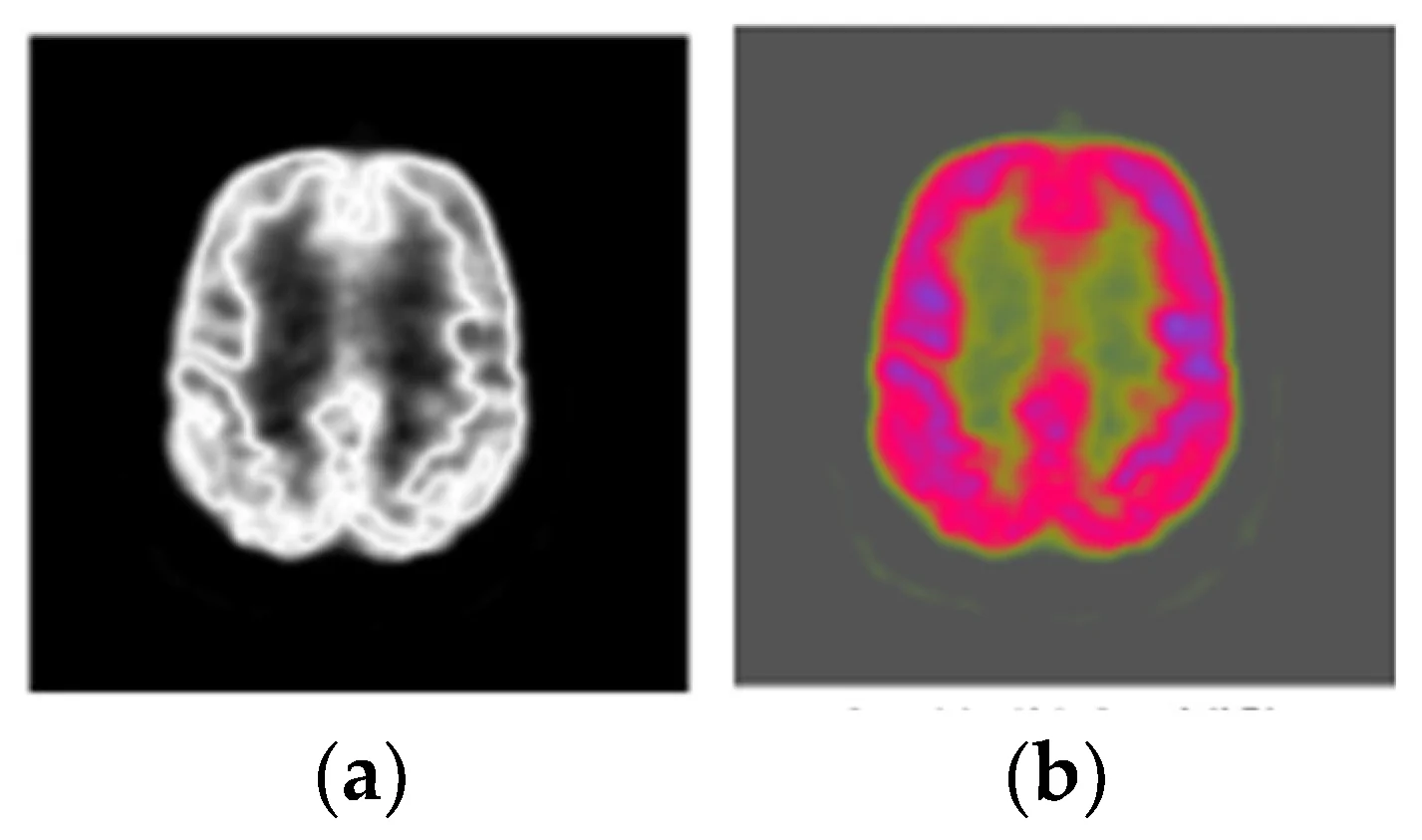
Color space conversion of the PET image: (**a**) Grayscale; (**b**) YUV.

In this paper, we consider wavelet-based fusion using Python with different input images. Aside from the raw images taken directly from the dataset, the other images also used as input images are presented in Figure 3. Namely, for our investigation, the original PET image from the dataset was additionally converted to grayscale (see Figure 3a) and to the YUV color space (see Figure 3b). The motivation for the conversions comes from the need to examine whether the color space representation impacts the quality of the PET-MRI fused image, and how those changes affect the end result using PyWavelets. The conversions were performed in Python using the corresponding procedure explained below.

The conversion of the RGB image to grayscale was performed by only extracting the luminance channel, computed as Y=0.299×R +0.587×G + 0.114×B [44]. While the conversion from RGB to YUV was performed by following the standard linear transformation:

Y = 0.299 ×R + 0.587×G + 0.114×B

U=−0.14713×R−0.28886×G+0.436×B

V=0.615×R−0.51499×G−0.10001×B

Here, Y is the luminance component, and U and V are known as chrominance components. A more detailed explanation of the YUV color space can be found in [45].

### 3.3. Python and PyWavelets

Python is an interpreted programming language widely used in diverse applications due to its versatility and comprehensive library support. Its interactive interpreter enables rapid experimentation and efficient exploration of the language features [46]. A case study presented in [47] illustrates Python’s capabilities in data processing and visualization, including a multimodal approach that combines magnetic resonance imaging (MRI) and electroencephalography (EEG) data for working memory assessment.

Python offers numerous packages for specialized tasks. In the present paper, we employ the PyWavelets package [48], which is suitable for our topic and provides n-dimensional discrete wavelet transforms and sets of predefined wavelets. The package supports one-, two-, and three- dimensional DWT, with results validated against MATLAB implementation. An orthogonal DWT implementation in Python is presented in [49], where the authors provide results closely matching those obtained with the PyWavelets module.

### 3.4. Compared Wavelet Families

A wide variety of wavelets can be selected for performing the wavelet transform. The Haar wavelet is the simplest discontinuous wavelet and closely resembles a step function, see [39]. The Daubechies wavelets are a family of wavelets introduced by Ingrid Daubechies to overcome the drawbacks of the Haar wavelet. Wavelets from the Daubechies family have compact support, are nonsymmetric (except one), and are orthogonal. Daubechies, biorthogonal wavelets, and coiflets are denoted with a number after their respective abbreviations: ‘db’, ‘bior’, and ‘coif’. The number denotes the order of the Daubechies wavelets, which is the same as the number of vanishing moments of the wavelet. For the biorthogonal wavelets, it is the number of respective vanishing moments for the reconstruction and decomposition filters. Finally, for the coiflets, it denotes the number of vanishing moments for both the scaling functions and the wavelet. The Daubechies ‘db1’ wavelet is the same as the Haar wavelet. Symmetry, linear phase property, biorthogonality, and compact support can be observed among the biorthogonal wavelets, which come in pairs, one wavelet for decomposition and one for reconstruction. Near-symmetry can be observed in the coiflets family of wavelets that also contain vanishing moments. The discrete Meyer wavelet also possesses the properties of orthogonality and symmetry; more about wavelet families can be found in [38,40].

**Figure 4:**
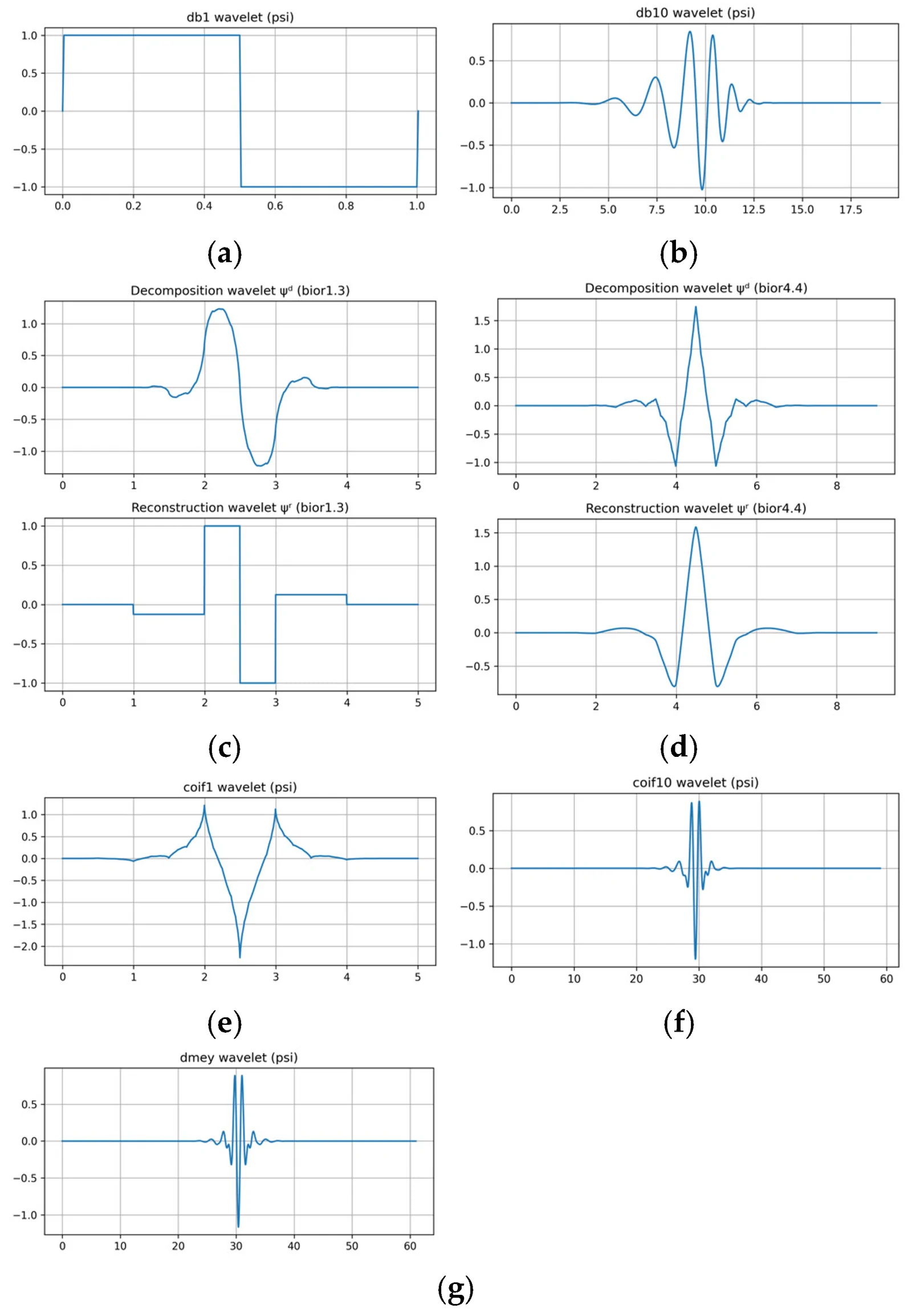
Wavelets: (**a**) ‘db1’; (**b**) ‘db10’; (**c**) ‘bior1.3’; (**d**) ‘bior4.4’; (**e**) ‘coif1’; (**f**) ‘coif10’; (**g**) ‘dmey’.

The PyWavelets module can be used to perform the wavelet transform in Python. PyWavelets is suitable for this transform because it contains several built-in wavelet families. Visual representation of the Daubechies (‘db1’ and ‘db10’), biorthogonal (‘bior1.3’ and ‘bior4.4’), coiflets (‘coif1’ and ‘coif10’), and discrete Meyer (‘dmey’) wavelets, which will be used in the experimental results, is given in Figure 4.

### 3.5. Implementation Details

The decomposition and the fusion were performed on a physical Windows 11 Pro (Microsoft Corporation, Redmond, WA, USA) Dell Vostro 3520 laptop (Dell Technologies Inc., Round Rock, TX, USA) with an x64-based Intel(R) Core(TM) i5-1235U (1.30 GHz) processor and Intel(R) UHD integrated graphics (Intel Corporation, Santa Clara, CA, USA), with 8 GB of installed RAM, and a 512 GB SSD. The version of the PyWavelets library used was PyWavelets 1.8.0, and the Python version was Python 3.11. The source code containing the scripts used for the decomposition, fusion, and quality metrics calculations is available in a GitHub repo provided in the Supplementary Materials section.

## 4. Results

In this section, we present the main results for the performed wavelet-based image decomposition and fusion of the medical images using Python and the PyWavelets library.

### 4.1. Results for Wavelet-Based Decomposition

The DWT is performed in Python using the dwt2 function for 2D discrete wavelet transform from the PyWavelets module, such that four sub-images: LL (approximation, low frequency), LH (horizontal features), HL (vertical features), and HH (diagonal features) of the original image are obtained. The LH, HL, and HH contain information about the high-frequency content or detection of the respective edges of the original image. They are obtained through filtering and down-sampling both horizontally and vertically. In other words, the approximation is obtained through low-pass filtering of both rows and columns of the image, and the detail is obtained through a combination of low-pass and high-pass filtering; see Figure 1 and refer to [50] for more details.

**Figure 5:**
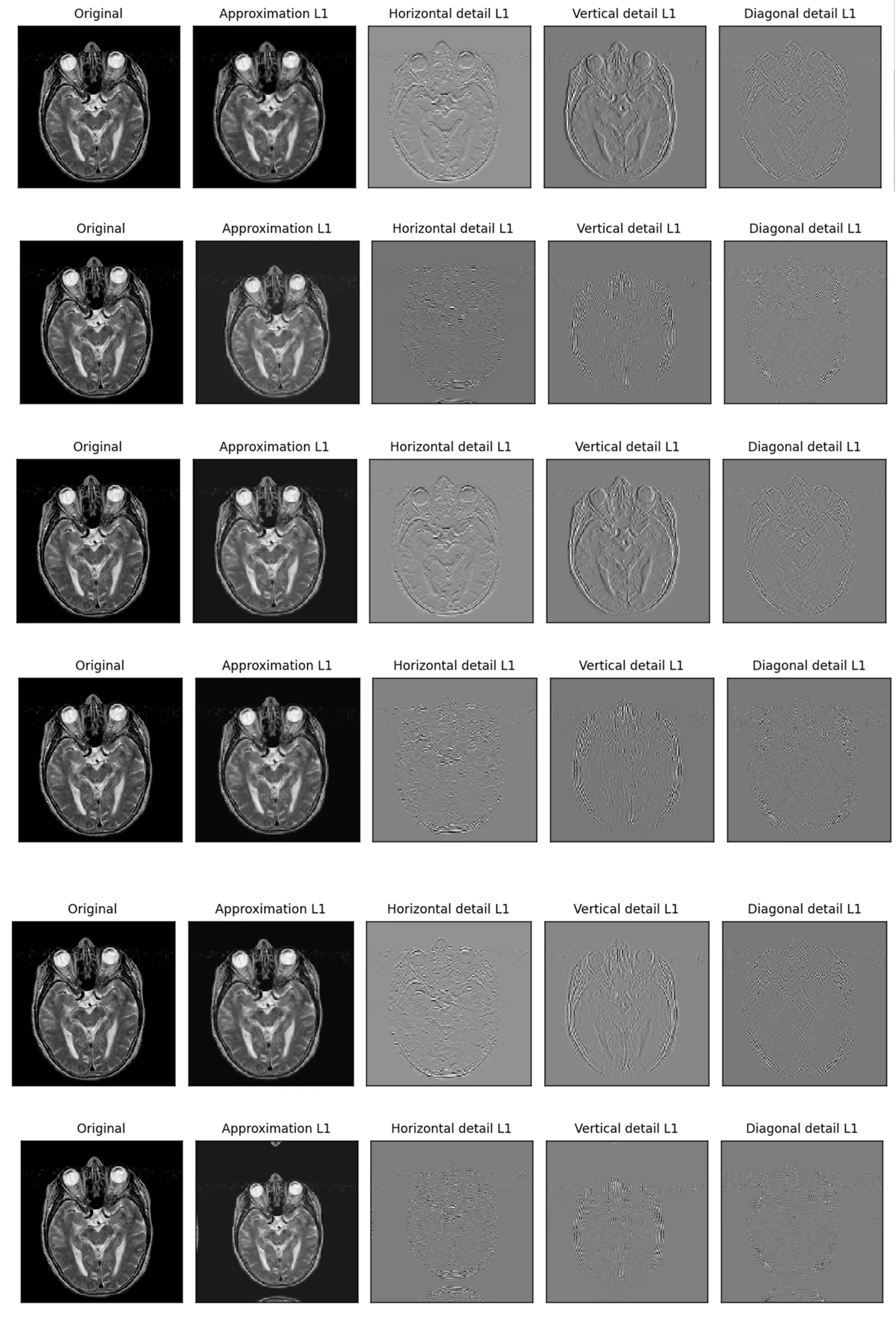

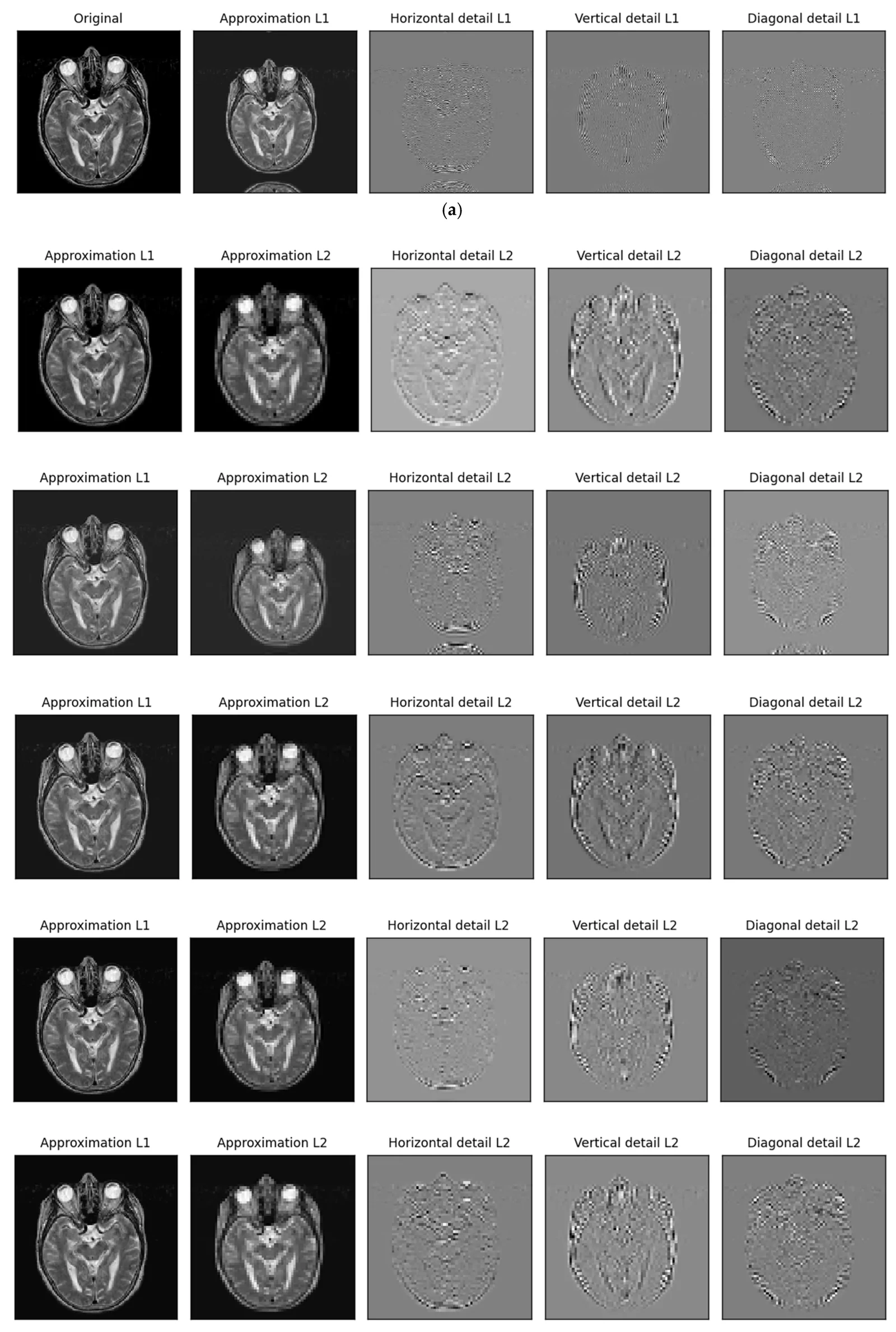

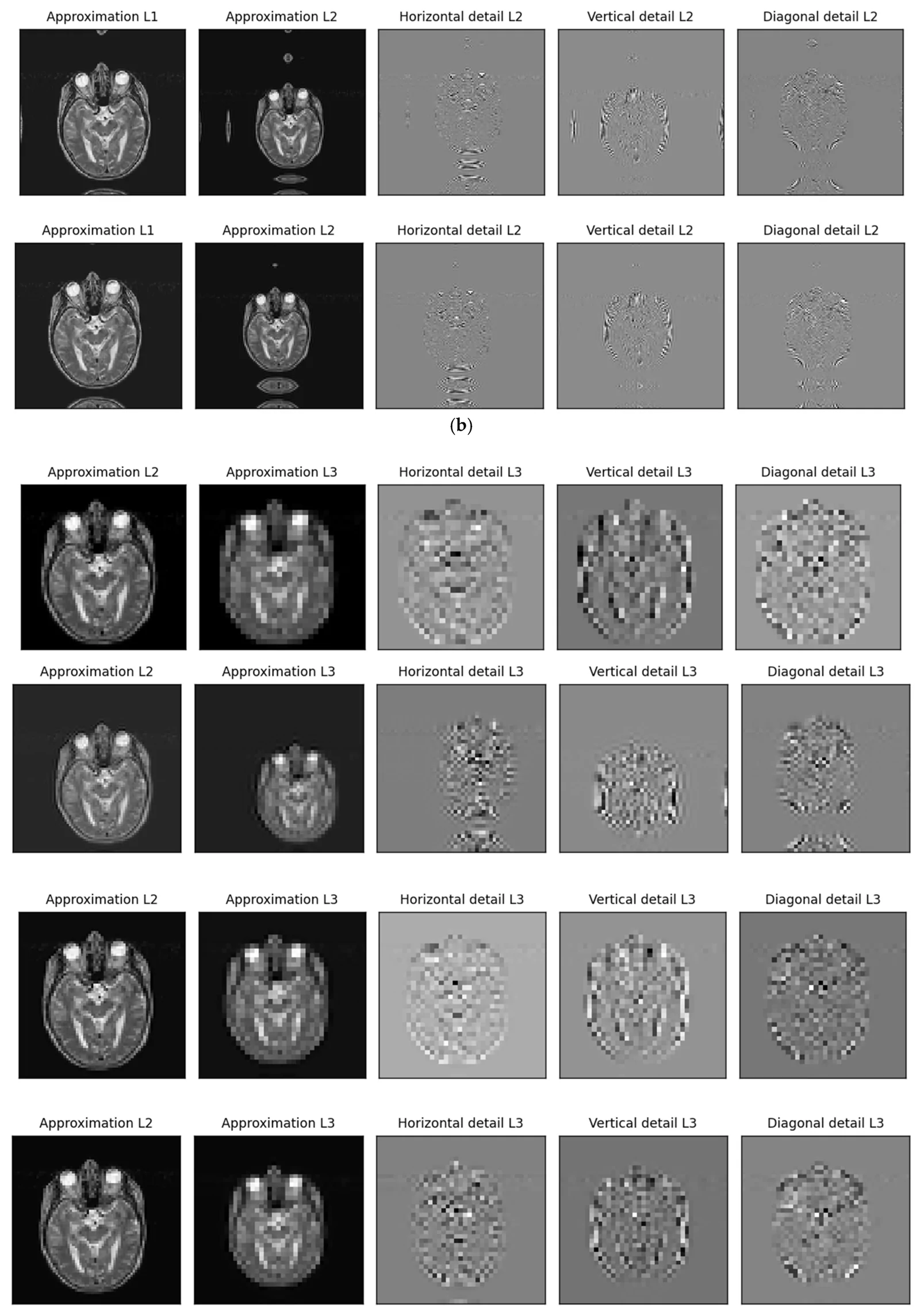

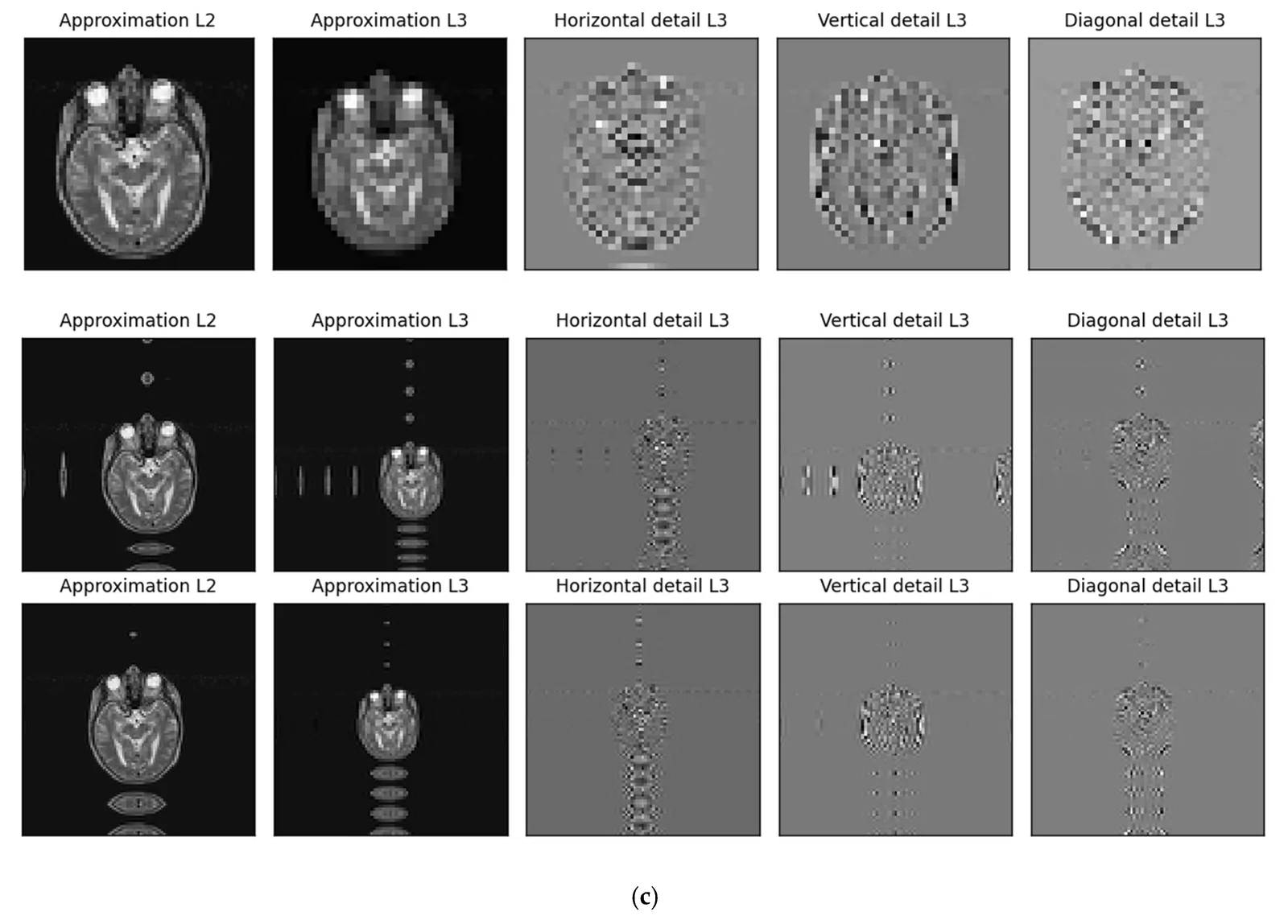
Comparison of the results obtained in the first, second, and third levels of decomposition of an MRI image in Python, using the corresponding wavelets: (**a**) First level using ‘db1’, ‘db10’, ‘bior1.3’, ‘bior4.4’, ‘coif1’, ‘coif10’, ‘dmey’, respectively; (**b**) Second level using ‘db1’, ‘db10’, ‘bior1.3’, ‘bior4.4’, ‘coif1’, ‘coif10’, ‘dmey’, respectively; (**c**) Third level using ‘db1’, ‘db10’, ‘bior1.3’, ‘bior4.4’, ‘coif1’, ‘coif10’, ‘dmey’, respectively.

In the present paper, wavelet-based decomposition using Python is performed on the images previously presented in Figure 2. Specifically, on the CT and MRI images (see Figure 2a,b), on the second MRI image with a different cross-section of the brain (Figure 2c), and on the PET image (see Figure 2d), while using several different wavelets given in Figure 4. Selected samples from the obtained results are presented in Figure 5 and the influence of the wavelet on the details obtained after wavelet decomposition is discussed. The results obtained for the first, second, and third level decompositions of an MRI image using the ‘db1’, ‘db10’, ‘bior1.3’, ‘bior4.4’, ‘coif1’, ‘coif10’, and ‘dmey’ wavelets are given respectively in Figure 5a–c. In the first level of decomposition, the differences in the results using different wavelets can be clearly seen, such that only a small difference may be noticeable in the comparison of the approximation sub-images. According to the presented results and the investigation that was done, it is obvious that a greater amount of information in the horizontal, vertical, and diagonal detail sub-image is obtained when using the ‘db1’ wavelet. The same conclusion can be drawn for the second level decomposition given in Figure 5b. Moreover, at this level, even bigger visible differences are evident in the approximation sub-images as well, and not only in the detail sub-images. The third level of decomposition, presented in Figure 5c, yields similar conclusions, but the images are very blurry and probably not very useful for this kind of application. We have decided to present them here to illustrate our findings for the third level of decomposition based on the examined images.

From the performed decompositions, one can conclude that the simpler wavelet, such as the ‘db1’, yields better results in comparison to a wavelet that contains more modulations (see the example results in Figure 5 for the other wavelets from Figure 4). We can also notice the appearance of distortion or artefacts in the second and third level decomposition when using the ‘coif1’, ‘coif10’, and ‘dmey’ wavelets, for the MRI and the CT image. Additionally, as expected, with further levels of decomposition, the approximation of the next level gets even more blurry due to the omission of high-frequency components. We obtained similar results for the wavelet-based decomposition process of the CT and the MRI image given in Figure 2a and d, respectively, but due to the extensiveness of the results, we decided not to provide them here. From the results for the CT and the MRI image, the same conclusion can be made.

**Figure 6:**
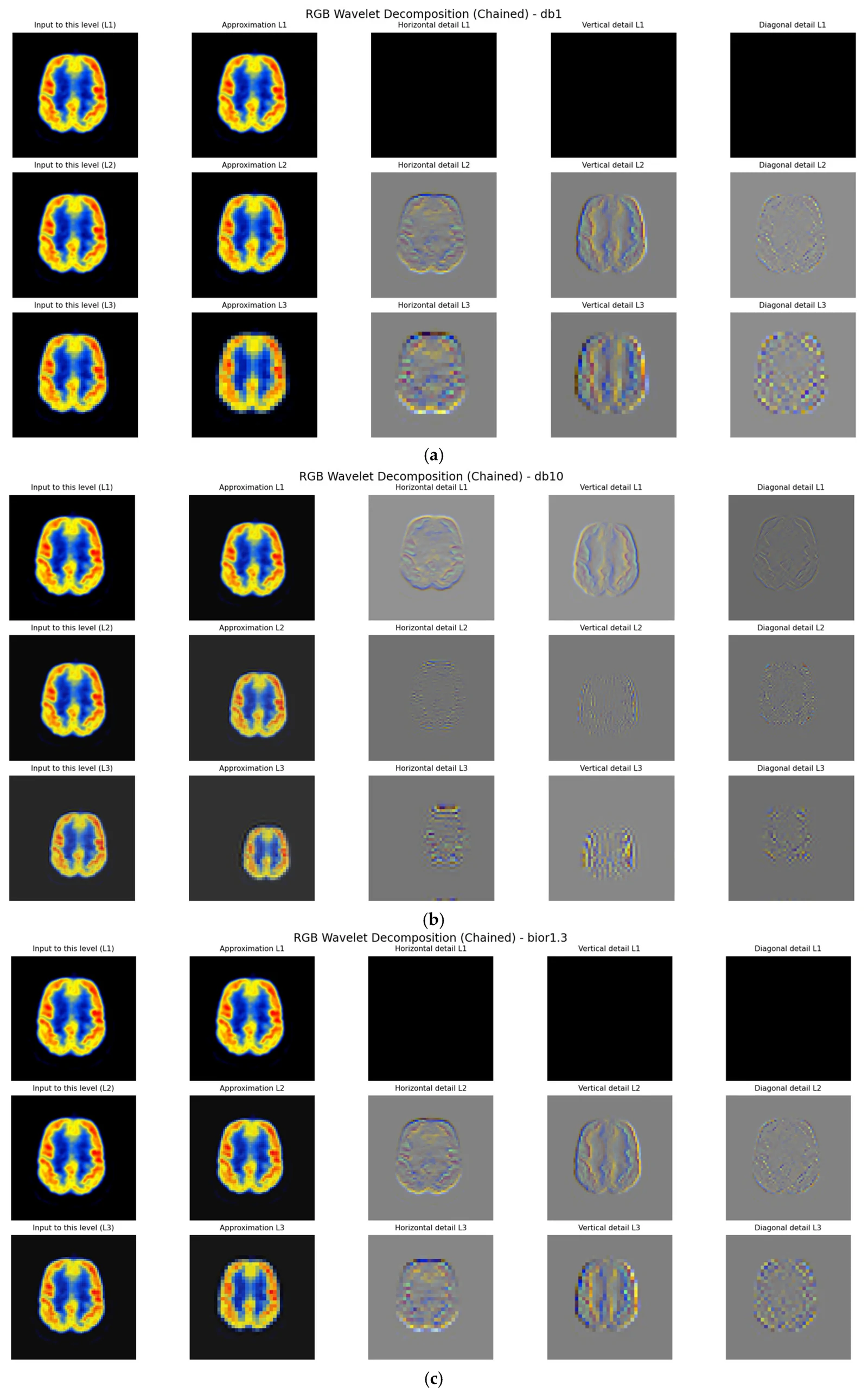

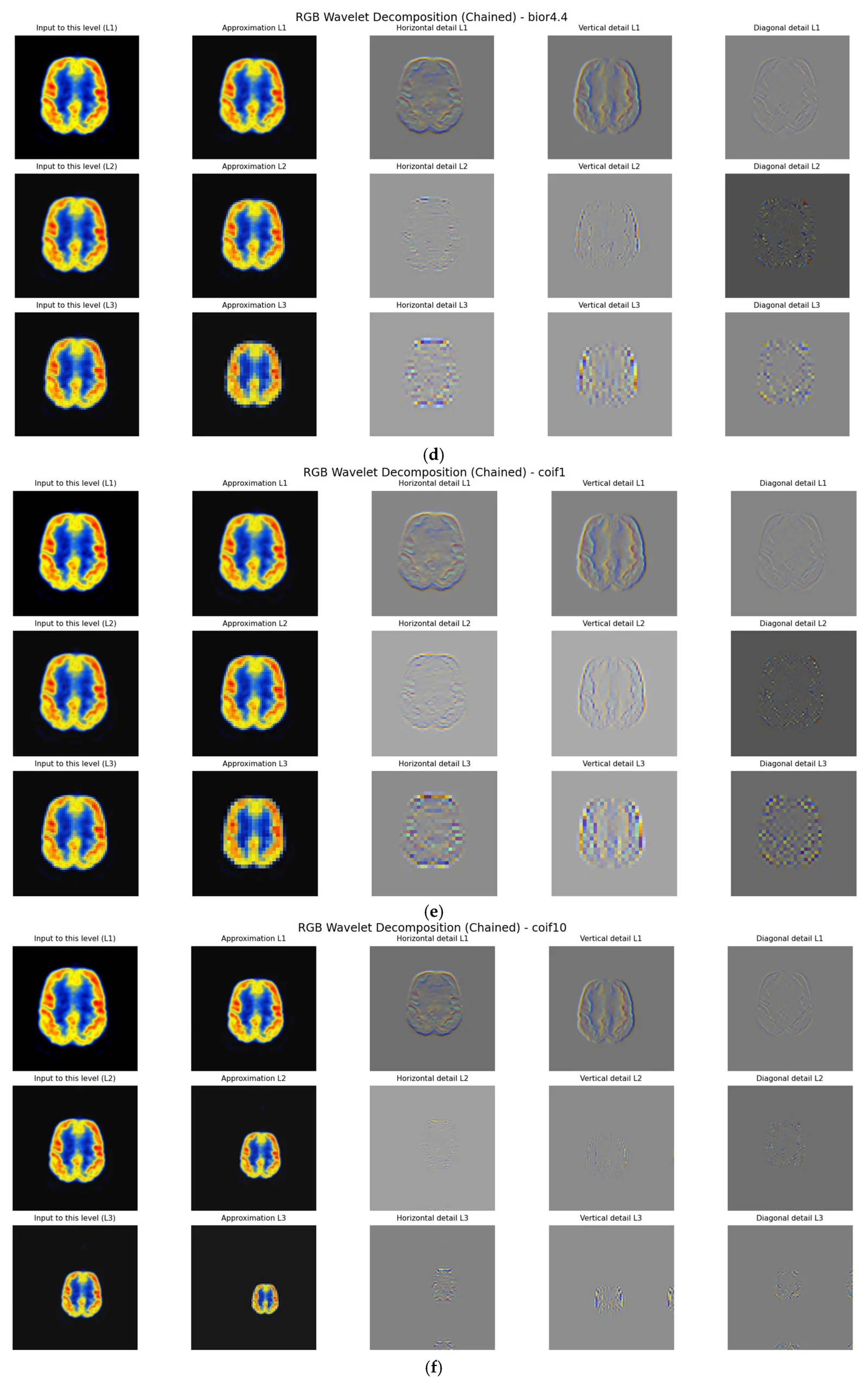

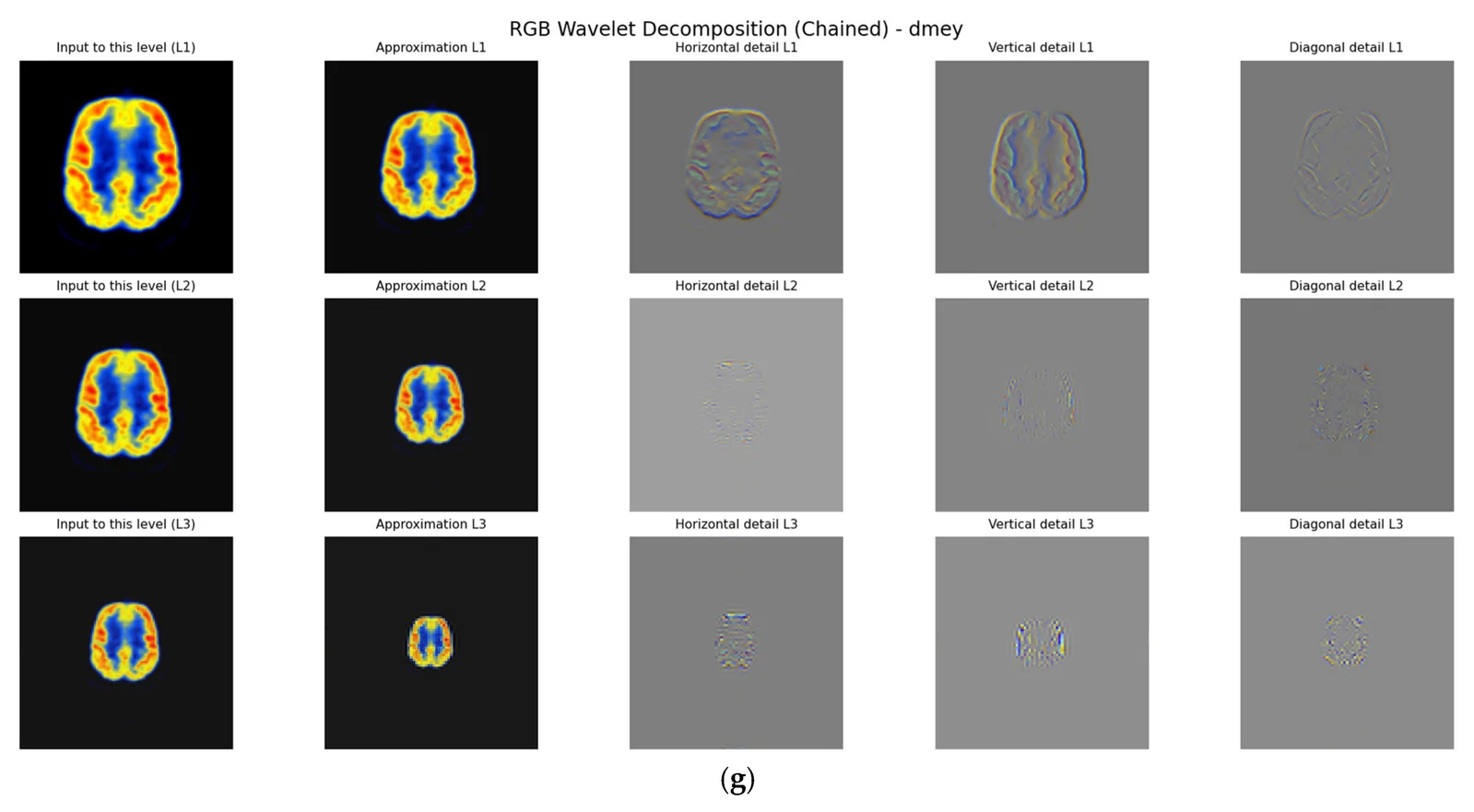
Comparison of the results obtained in the first, second, and third levels of decomposition of a PET image in Python, using the corresponding wavelets: (**a**) ‘db1’; (**b**) ‘db10’; (**c**) ‘bior1.3’; (**d**) ‘bior4.4’; (**e**) ‘coif1’; (**f**) ‘coif10’; (**g**) ‘dmey’.

Figure 6 presents selected results for the wavelet-based decomposition of the PET image given in Figure 2c. The results for the first, second, and third levels of wavelet-based decomposition of the PET image using ‘db1’, ‘db10’, ‘bior1.3’, ‘bior4.4’, ‘coif1’, ‘coif10’, and ‘dmey’ wavelets are given.

From the obtained results in Figure 6, we can conclude that the results are opposite to the results in Figure 5, which means that when simpler wavelets such as ‘db1’ and ‘bior1.3’ are used, the horizontal, vertical, and diagonal details are not noticeable in the first level, whereas in the second and third levels, they are visible. On the other hand, when we use wavelets with modulations, such as ‘db10’, ‘bior4.4’, ‘coif1’, ‘coif10’, and ‘dmey’, in the first level, we have information for the horizontal details, vertical details, and diagonal details, but in the second level the information is less, and in the third level it is even less or does not exist. The opposite results are probably due to the greater high-frequency content in the color image compared with the grayscale image.

In this paper, the terms sub-images and sub-bands are both used to capture two complementary perspectives of the 2D DWT. Specifically, the term sub-images is used for the spatial representation of the decomposition, while the term sub-bands is used for the frequency-domain interpretation and analysis of the decomposition.

**Figure 7:**
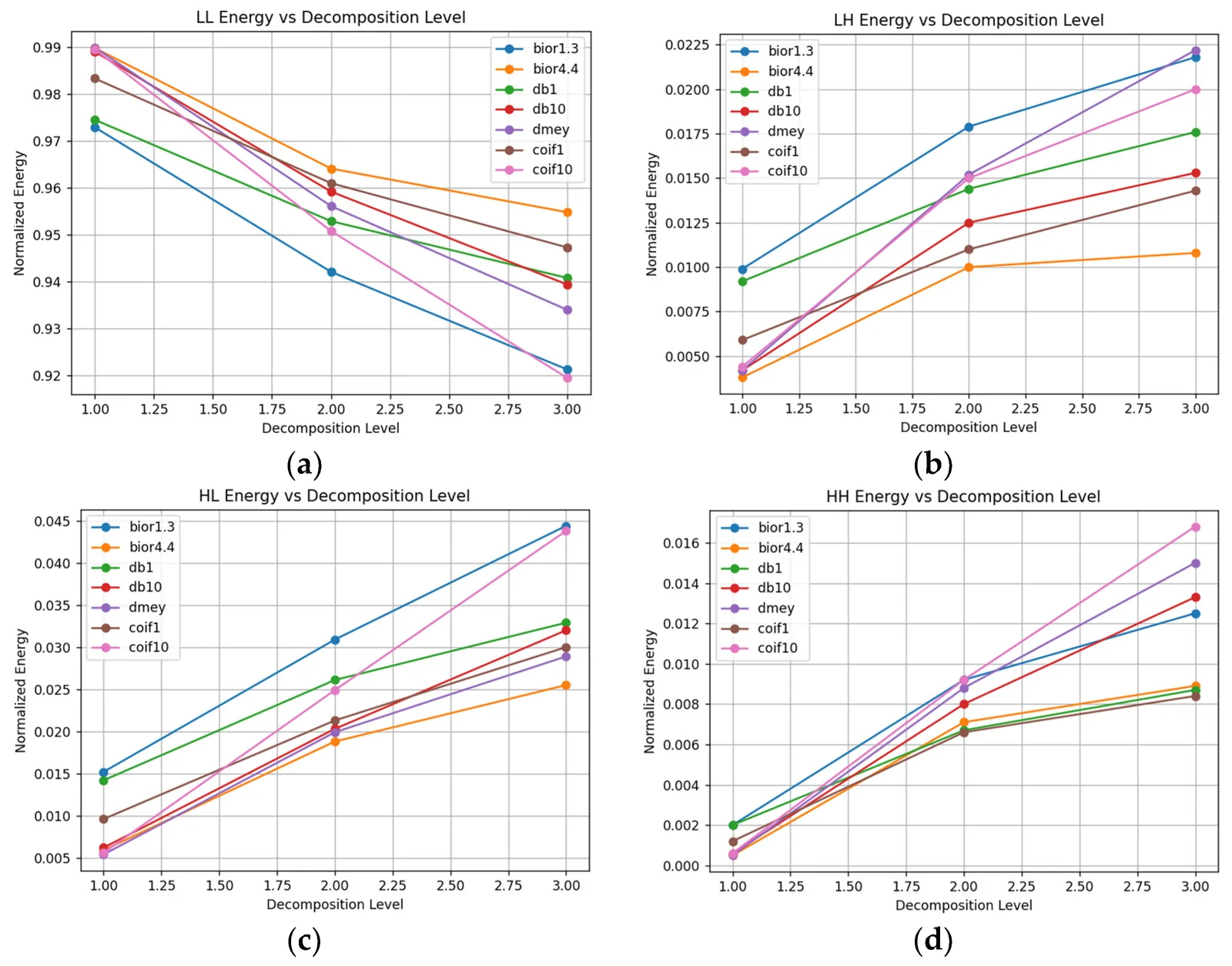
Comparison of coefficient energy vs. the decomposition level for the MRI image decomposition with different wavelets: (**a**) Energy of the LL (approximation coefficients); (**b**) energy of the LH (horizontal features) detail coefficients; (**c**) energy of the HL (vertical features) detail coefficients; (**d**) energy of the HH (diagonal features) detail coefficients.

**Figure 8:**
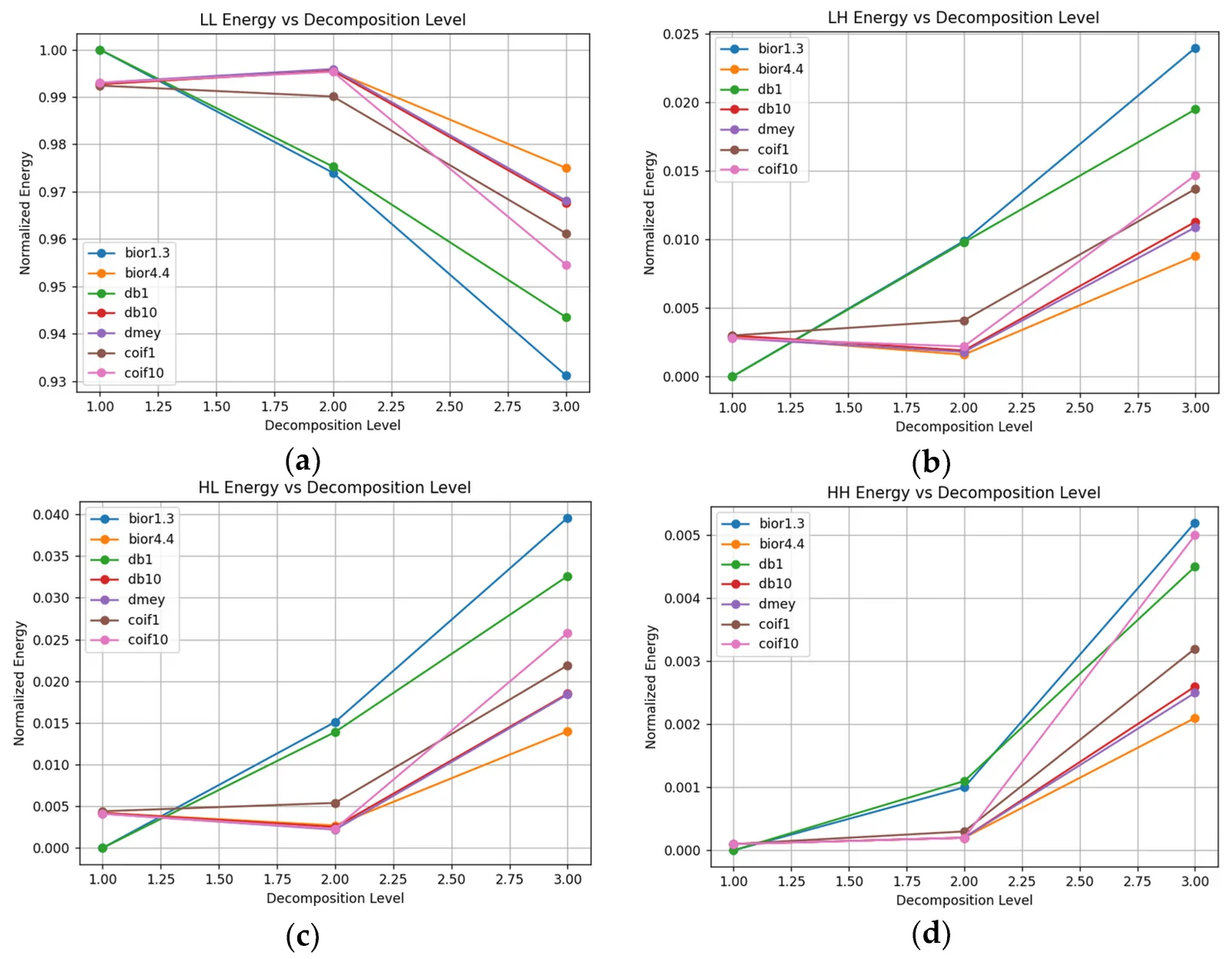
Comparison of coefficient energy vs the decomposition level for the PET image decomposition with different wavelets: (**a**) Energy of the LL (approximation coefficients); (**b**) energy of the LH (horizontal features) detail coefficients; (**c**) energy of the HL (vertical features) detail coefficients; (**d**) energy of the HH (diagonal features) detail coefficients.

The quality of the decomposition was additionally quantitatively assessed through the energy of coefficients. This parameter, defined as the sum of squared wavelet coefficients at each decomposition level, reflects the distribution of signal information across the sub-bands [51]. In that manner, the total wavelet energy, which accounts for all sub-bands (LL, LH, HL, HH), was calculated, and the normalized values of each sub-band energy by the total energy are presented in Table 1 for the MRI image decomposition, and in Table 2 for the PET image decomposition, the data from which are visualized in the plots shown in Figure 7 and Figure 8, respectively.

As can be expected from multiresolution decomposition, Figure 7 demonstrates a decrease in the energy of the approximation coefficients and, correspondingly, an increase in the energy of the detail coefficients across the three decomposition levels. This figure illustrates the trade-offs involved in preserving the approximation versus preserving the details. For example, it is evident from Figure 7a that ‘bior4.4’ retains more information in the approximation coefficients compared with the other wavelets. Consequently, in Figure 7b, ‘bior4.4’ has weak growth and lower values for the detail coefficients’ energy. A practical application of this finding could be the use of ‘bior4.4’ for noise suppression; however, there remains a risk of missing some finely detailed information. An example where the ‘bior4.4’ filter is used within an image denoising algorithm can be found in [52].

Similar to Figure 7, Figure 8 shows the results of the PET image decomposition with respect to the energy of the coefficients across the three decomposition levels. Again, it can be noted that ‘bior4.4’ retains the highest values for the energy of the approximation coefficients up to the third level of decomposition. From Figure 8a–c, it is clear that ‘bior1.3’ performs best in preserving the detail coefficients’ energy.

### 4.2. Results for Wavelet-Based Fusion

Our first goal is to find which basic fusion rule: max, min, or average, is more appropriate for the given fusion task. The second goal is to find which wavelet is more suitable for wavelet-based fusion.

Therefore, we performed fusion on the CT and MRI images (given in Figure 2a,b), the PET grayscale and MRI images (given in Figure 2d and Figure 3a), the original PET and MRI images (given in Figure 2c,d), and the PET YUV and MRI images (given in Figure 2d and Figure 3b). The fusion was performed with all selected wavelets as in the decomposition part (‘db1’, ‘db10’, ‘bior1.3’, ‘bior4.4’, ‘coif1’, ‘coif10’, ‘dmey’).

We obtain fused images in the first, second, and third levels and, moreover, we obtain the corresponding quantitative image quality evaluation by entropy, standard deviation, and mutual information. Namely, entropy and mutual information (MI) values were computed on 8-bit intensities. Entropy was calculated as base-2 Shannon entropy of the fused image from the 256-bin histogram, while MI calculations used base-2 logarithms on a 256 × 256 joint histogram. For the colored images, the metrics were computed per channel and averaged over the R, G, and B channels.

Due to the volume of fused images obtained during the experiments, we present only those images that helped to make the conclusions for the aforementioned goals.

**Figure 9:**
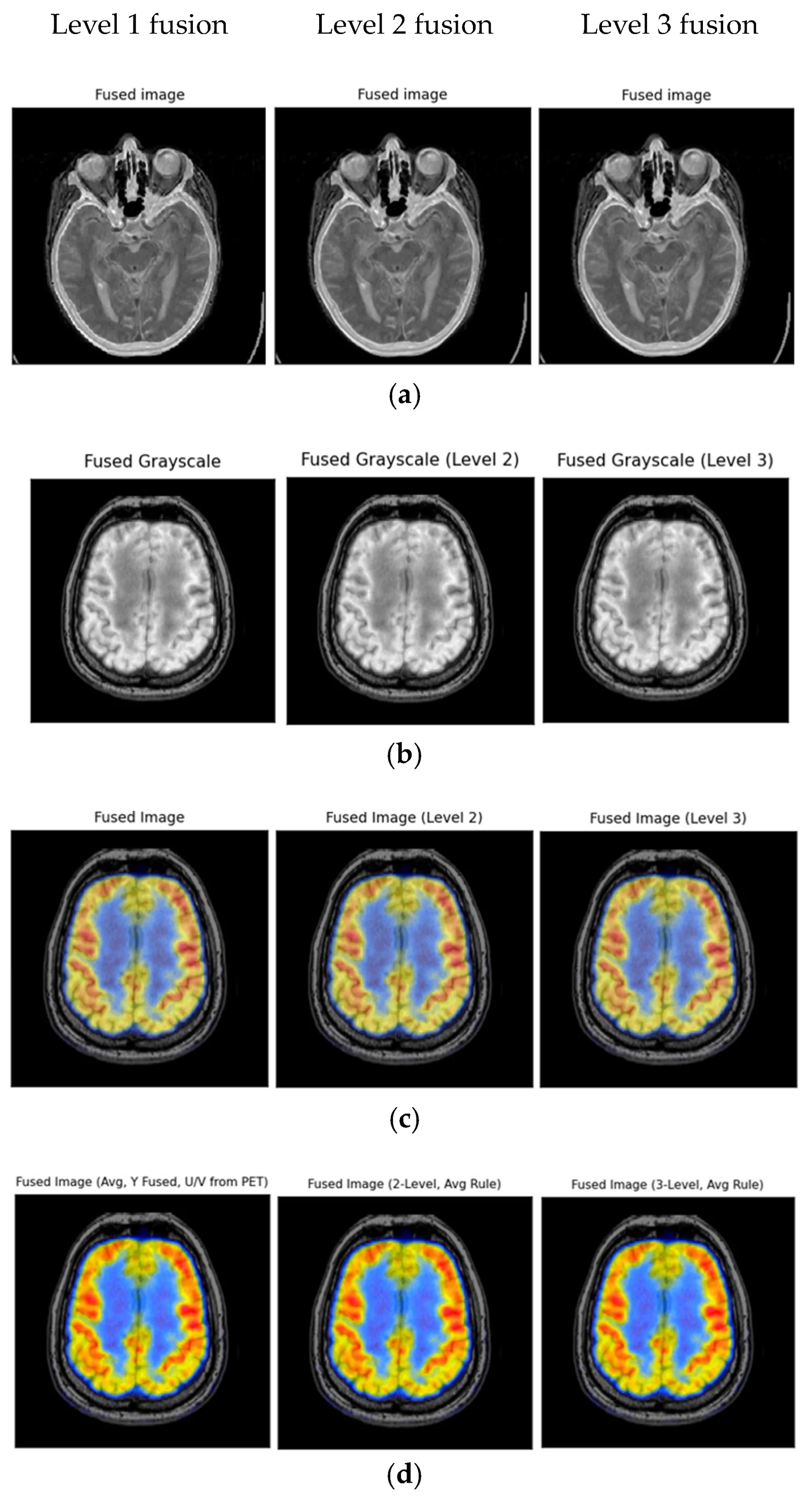
Comparison of one-, two-, and three-level fusion results, using the average fusion rule with the ‘bior4.4’ wavelet: (**a**) CT and MRI; (**b**) PET grayscale and MRI; (**c**) PET without conversion and MRI; (**d**) PET YUV conversion and MRI.

Based on the results obtained for the max, min, and average fusion images in all three levels, we conclude that, for the examined images, the average rule yields the most favorable results for the quality of the fused image. Using the min fusion rule, it seems some information is lost. In contrast, when using the max fusion rule, the fused image seems to contain a redundant amount of information, making it very blurry. Hence, in Figure 9, the obtained results for the average rule are presented only for the ‘bior4.4’ wavelet.

**Figure 10:**
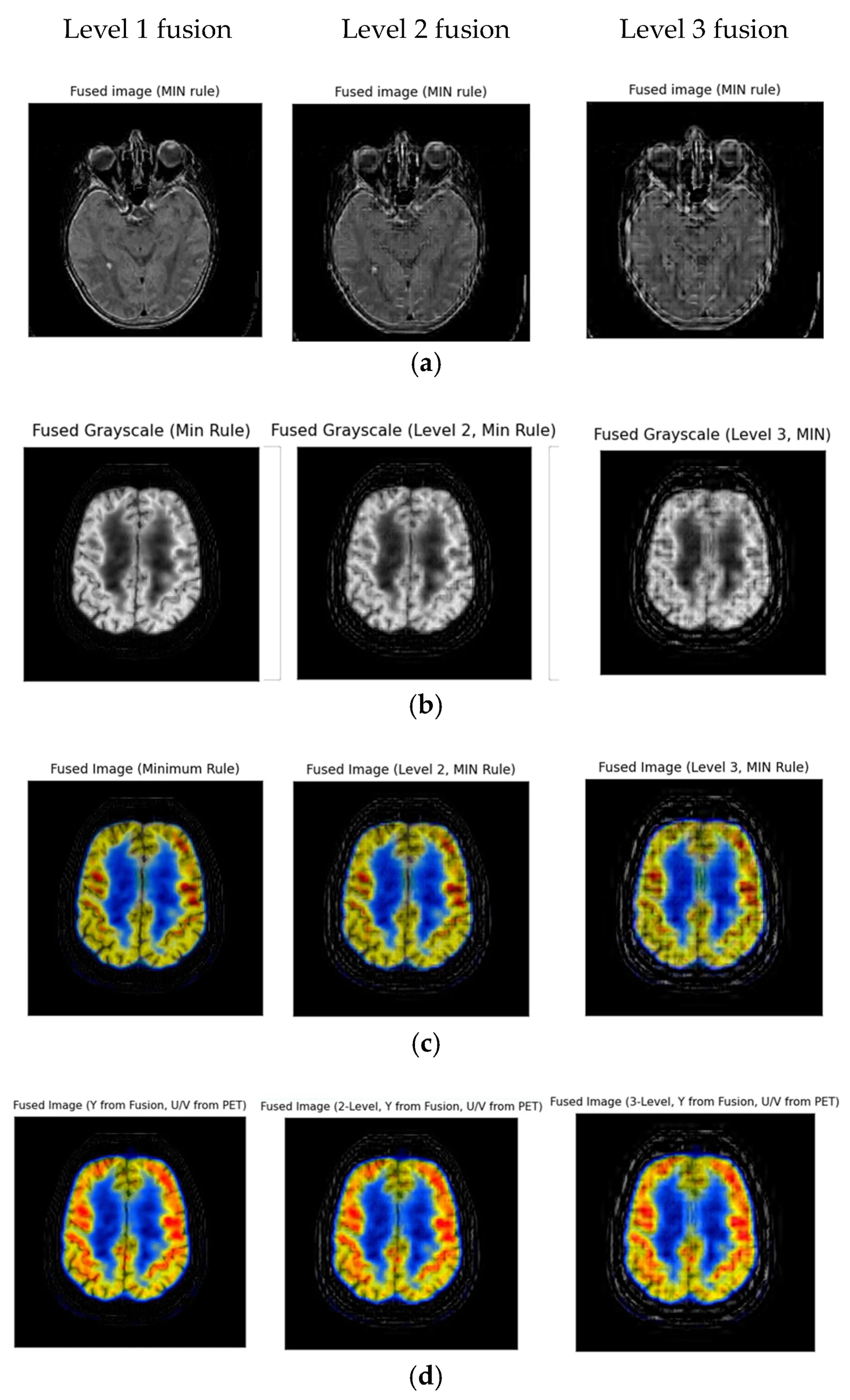
Comparison of one-, two-, and three-level fusion results, using the minimum fusion rule with the ‘bior4.4’ wavelet: (**a**) CT and MRI; (**b**) PET grayscale and MRI; (**c**) PET without conversion and MRI; (**d**) PET YUV conversion and MRI.

It should be noted that the maximum fusion rule approach yields even higher entropy values. However, it is clear from Figure 10 that lines appear in the fused image, especially in the three-level fusion. Whereas in Figure 9, the fused image is clearly visible, without any visually redundant information.

**Figure 11:**
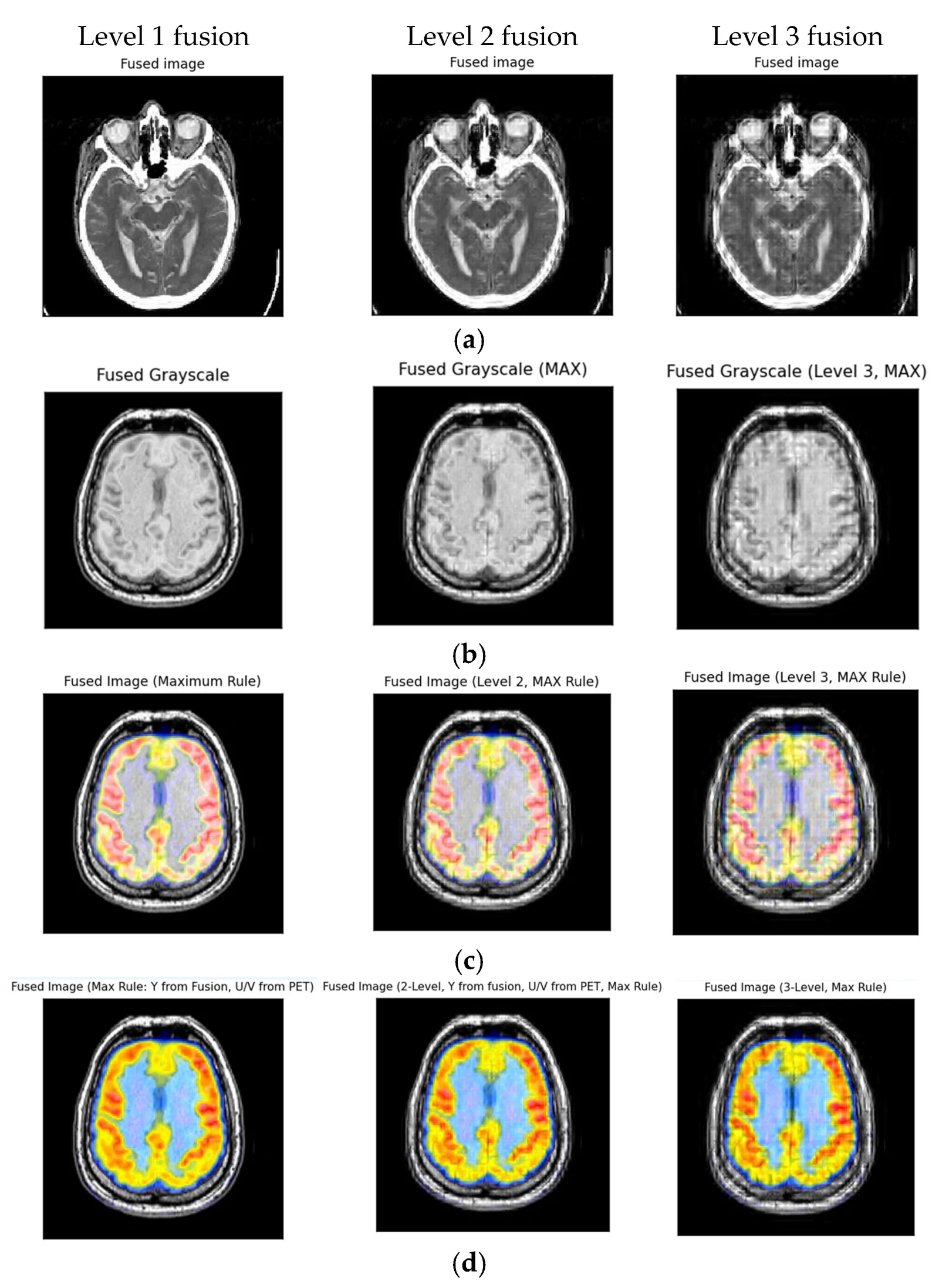
Comparison of one-, two-, and three-level fusion results, using the maximum fusion rule with the ‘bior4.4’ wavelet: (**a**) CT and MRI; (**b**) PET grayscale and MRI; (**c**) PET without conversion and MRI; (**d**) PET YUV conversion and MRI.

Similar results can be seen in Figure 11, where the fusion results obtained while using the maximum fusion rule are presented. Clearly, there are lines appearing in the fused image, especially in the third level.

**Figure 12:**
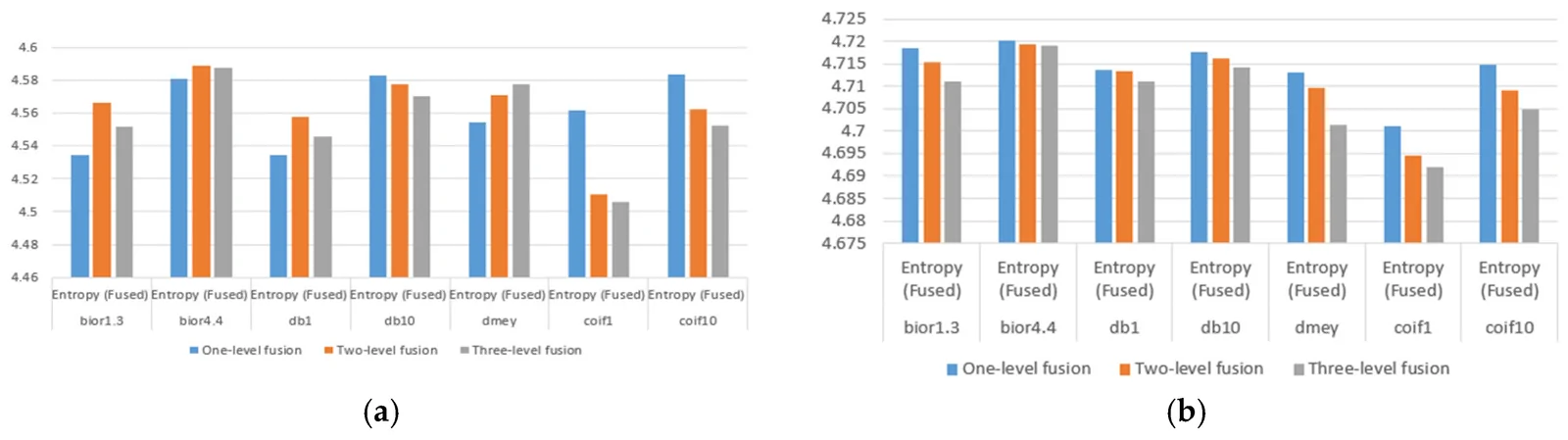

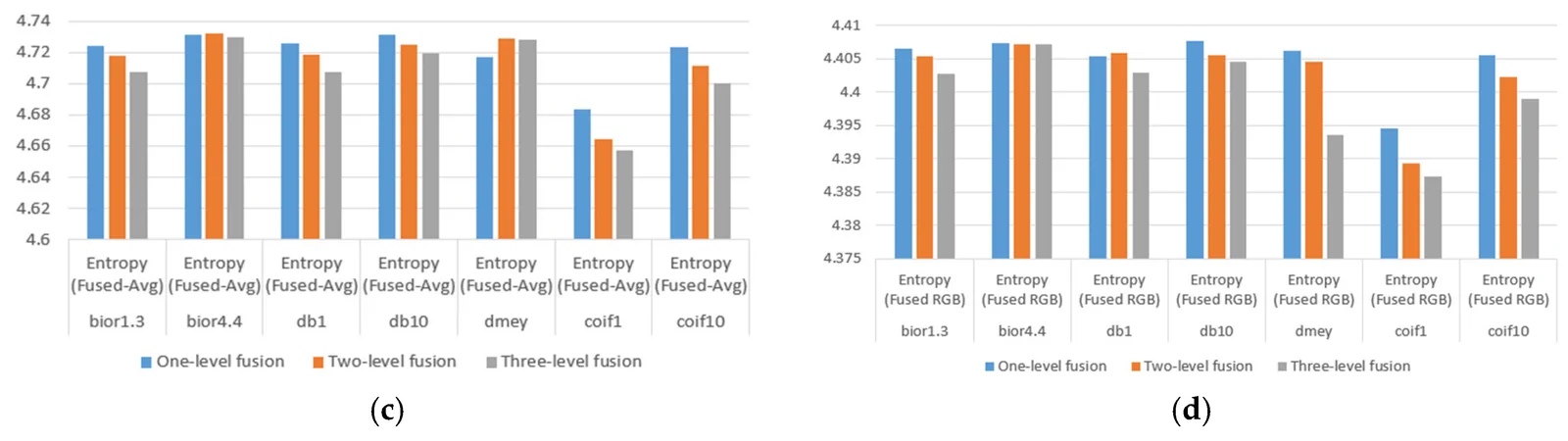
Comparison of entropy values from fusion using the average fusion rule: (**a**) CT and MRI; (**b**) PET grayscale and MRI; (**c**) PET without conversion and MRI; (**d**) PET YUV and MRI.

In Figure 12, we provide the results obtained for the entropy in all three levels for the same input images, using the average fusion rule, for all chosen wavelets, to conclude which wavelet is convenient for wavelet fusion.

From the charts presented in Figure 12, it can be seen that using the average fusion rule, the highest values for the entropy are consistently obtained using the ‘bior4.4’ wavelet, i.e., (a) 4.5887 for CT and MRI with two-level fusion, (b) 4.7202 for PET with grayscale conversion and MRI with one-level fusion, (c) 4.7320 for PET without conversion and MRI with two-level fusion, and (d) 4.4073 for PET with YUV conversion and MRI image with one-level fusion. If we compare these values, we can conclude that the PET without conversion and MRI approach yields the highest value. Our conclusion is that the entropy is highest for ‘bior4.4’ that this wavelet is best among the examined wavelets for the fusion of the examined images. The family of biorthogonal wavelets is biorthogonal, symmetric, has a linear phase property, and compact support, as per Section 3.4 or [38]. The choice of the wavelet seems to have a bigger impact on the values than the level of fusion.

Regarding the average fusion rule, for the chart in Figure 12, it can also be concluded that the entropy values for one- and/or two-level fusion are almost always higher than those for three-level fusion across all examined wavelets and image modalities (except for ‘dmey’ for the CT and MRI fusion). This could indicate a potential information loss when performing three-level fusion rather than introducing any improvements, and favors the sufficiency of two-level fusion.

For the same Figure 12, it is also noticeable that both the PET grayscale and the original PET images, fused with the MRI image, hold higher values for the entropy than the PET image converted to YUV color space.

**Figure 13:**
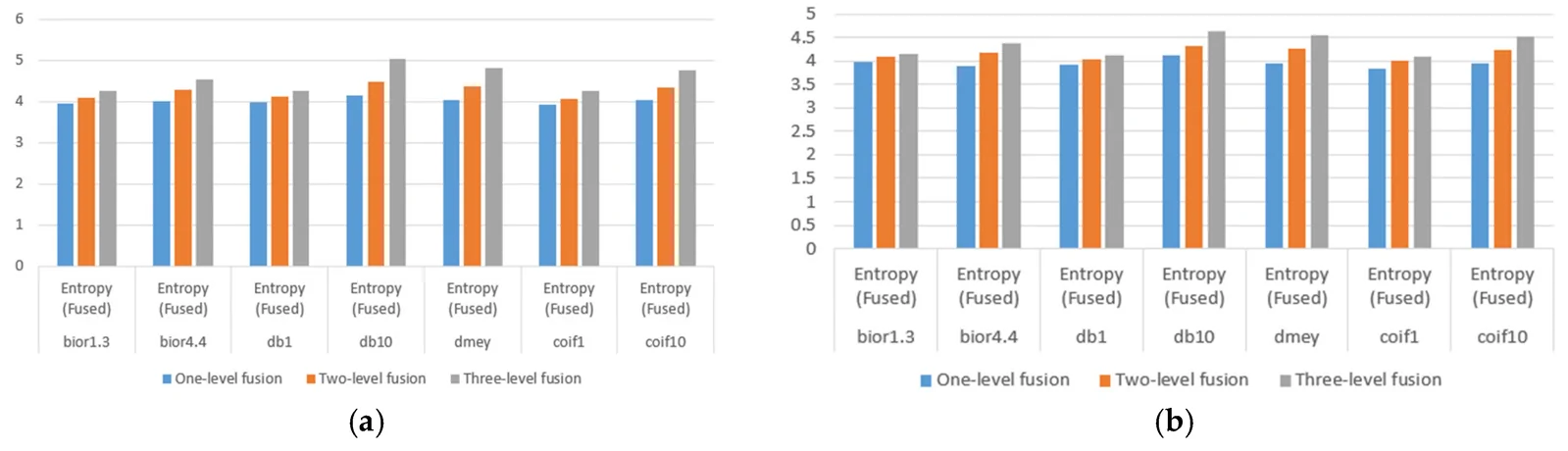

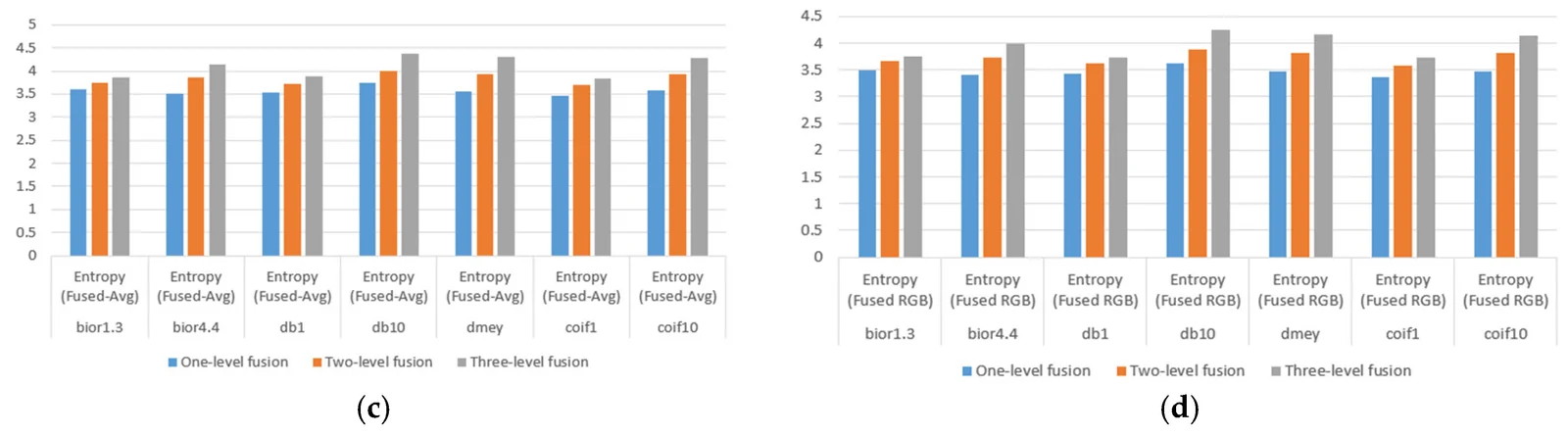
Comparison of entropy values from fusion using the minimum fusion rule: (**a**) CT and MRI; (**b**) PET grayscale and MRI; (**c**) PET without conversion and MRI; (**d**) PET YUV and MRI.

**Figure 14:**
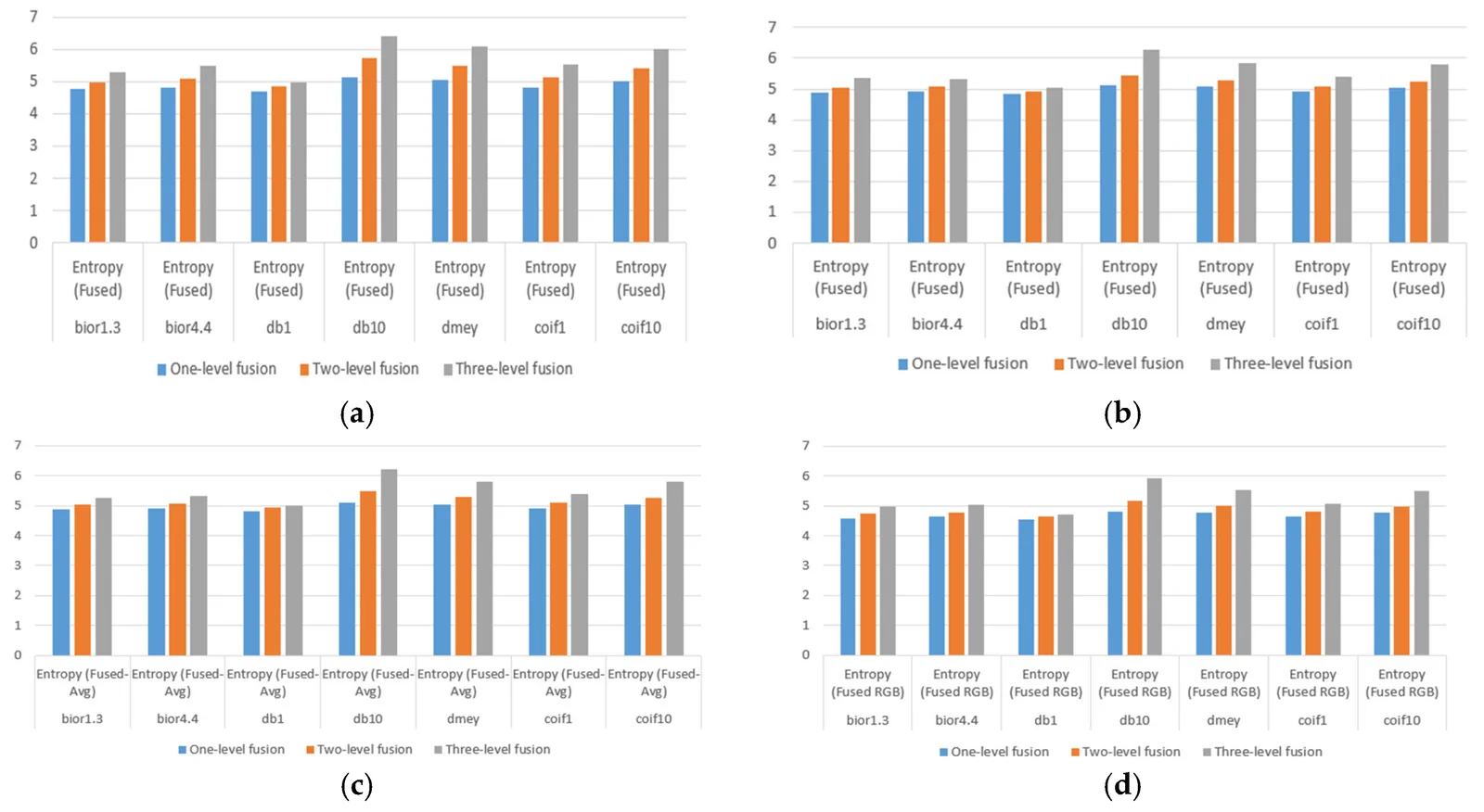
Comparison of entropy values from fusion using the maximum fusion rule: (**a**) CT and MRI; (**b**) PET grayscale and MRI; (**c**) PET without conversion and MRI; (**d**) PET YUV and MRI.

Regarding the minimum and maximum fusion rules, the charts presented in Figure 13 and Figure 14 indicate that the entropy value always rises with the level of fusion. The Daubechies ‘db10’ wavelet performs best in terms of the highest entropy values across both fusion rules. However, the high quantitative values of the entropy alone cannot justify the degradation in visual perception of the image previously presented in Figure 10 and Figure 11, where lines start to appear in the fused image.

**Figure 15:**
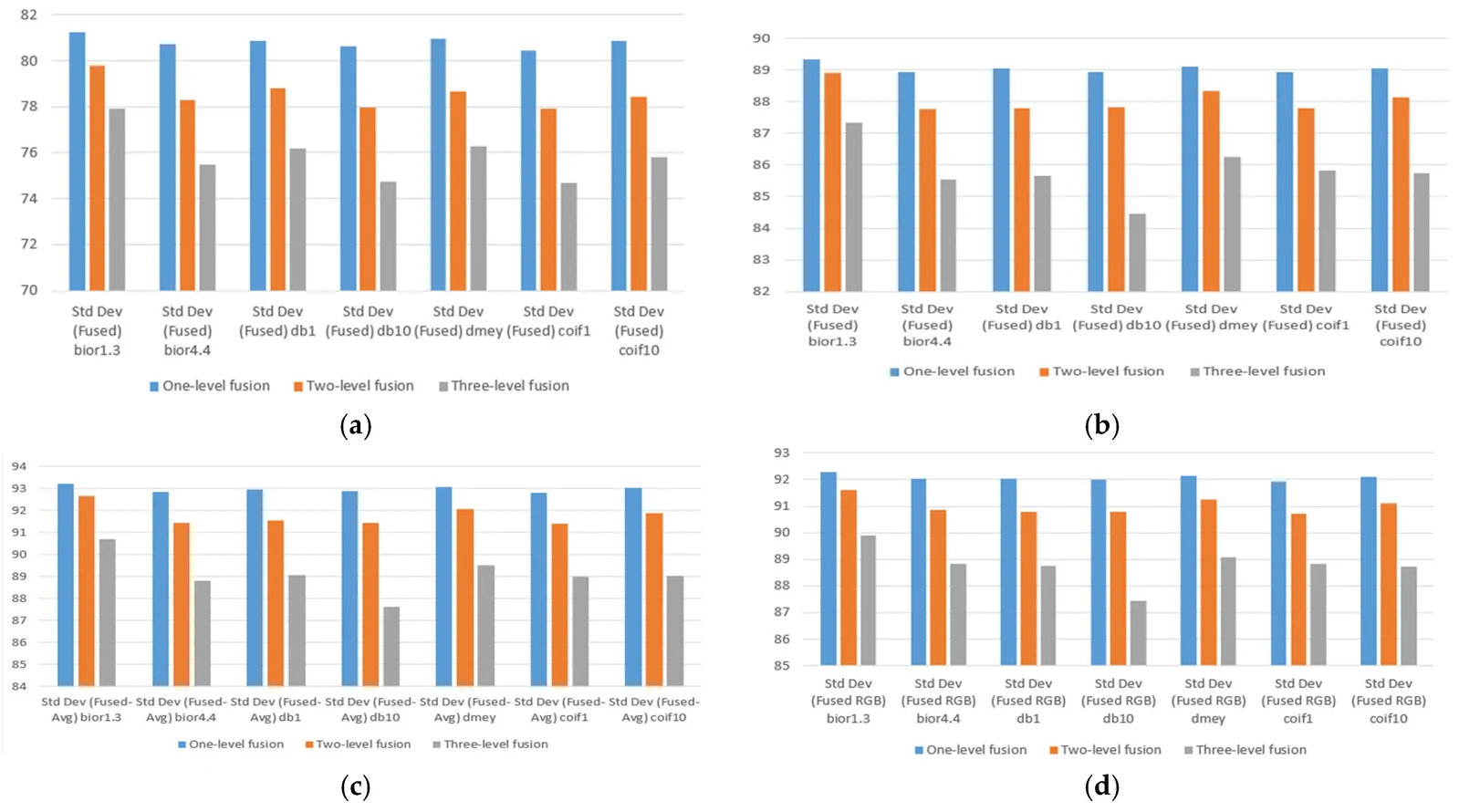
Comparison of standard deviation values from fusion using the maximum fusion rule: (**a**) CT and MRI; (**b**) PET grayscale and MRI; (**c**) PET without conversion and MRI; (**d**) PET YUV and MRI.

As shown in the charts presented in Figure 15, the highest values for standard deviation (Std Dev) are obtained for one-level fusion performed with the maximum fusion rule while using the ‘bior1.3’ wavelet. The highest values for the standard deviation are (a) 81.2213 for the fusion of the CT and MRI images, (b) 89.3366 for PET grayscale conversion and MRI fusion, (c) 93.2046 for fusion of the PET image without prior conversion and MRI, and (d) 92.2738 for PET YUV conversion and MRI fusion. Evidently, the highest of these values is obtained without prior conversion of the PET image.

The standard deviation values obtained from the fusion when using the minimum and the average fusion rules are lower and can be found in the charts presented as part of the Supplementary Materials (Figures S1).

The fused images in the three levels, with fusion performed with the maximum fusion rule and while using the ‘bior1.3’ wavelet, can also be found as part of the Supplementary Materials (Figure S1 FusionMAXRule). From the same figure, it can be seen that the results in the one-level fusion are also visually clearer than the two-level and three-level fusion results, because visible lines are appearing in the fused image, especially in the three-level fusion with the maximum rule. Fusion has also been performed with the minimum fusion rule and the average fusion rule. The results can be found in the Supplementary Materials (Figure S1 FusionMINRule and Figure S1 FusionAVGRule).

**Figure 16:**
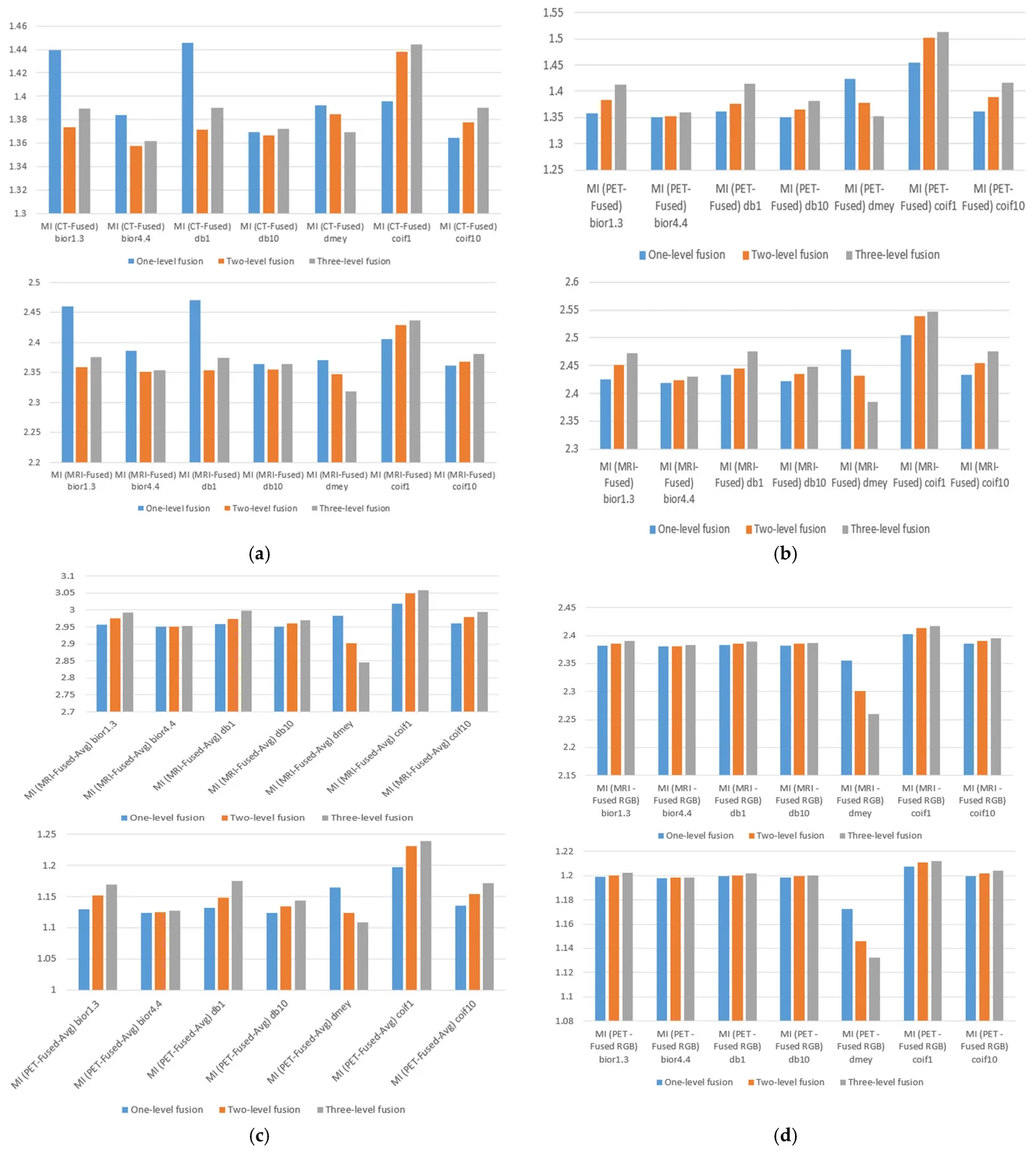
Comparison of mutual information (MI) values from fusion using the average fusion rule: (**a**) CT-Fused and MRI-Fused (CT and MRI fusion); (**b**) PET-Fused and MRI-Fused (PET grayscale and MRI fusion); (**c**) PET-Fused and MRI-Fused (PET without conversion and MRI fusion); (**d**) PET-Fused and MRI-Fused (PET YUV and MRI fusion).

Additionally, the values for the mutual information (MI) obtained when using the average fusion rule are also presented in the charts in Figure 16. Regarding the fusion of the CT and MRI images, it can be concluded that MI (CT-Fused) (1.4454) and MI (MRI-Fused) (2.4699) are highest for one-level fusion with the average fusion rule when the ‘db1’ wavelet is used. However, for the PET grayscale and MRI fusion, MI (PET-Fused) is actually highest (1.5232) for one-level fusion with the minimum fusion rule when the ‘db1’ wavelet is used, while MI (MRI-Fused) is highest (2.5473) for the three-level fusion with the average fusion rule when the ‘coif1’ wavelet is used. Looking at the PET without conversion and MRI fusion, MI (MRI-Fused) (3.0575) and MI (PET-Fused) (1.2397) are highest for three-level fusion with the average fusion rule when the ‘coif1’ wavelet is used. Finally, for the PET YUV and MRI fusion, MI (MRI-Fused) is highest (2.4168) for three-level fusion with the average fusion rule when the ‘coif1’ wavelet is used and MI (PET-Fused) is highest (1.3148) for three-level fusion with the minimum fusion rule when the ‘db1’ wavelet is used. The respective charts from the minimum and maximum fusion rule are given in the Supplementary Figure S2 (given in four parts for better visibility: Figure S2a–d).

Additionally, an ANOVA test was conducted for the PET without conversion and MRI fusion with the average fusion rule. The outcomes of the tests for both the one-way and repeated-measures ANOVA indicate that for the average fusion of said modalities, and specifically for the images we have used, the decomposition level does not significantly affect the standard deviation and the mutual information. Only entropy showed a significant effect (although with a small magnitude), and only in the repeated-measures analysis. For comparison, the ANOVA test was also performed for the PET without conversion and MRI fusion with the maximum fusion rule. In that case, entropy was significant in both one-way and repeated-measures tests, rising significantly in the third level of decomposition, while the standard deviation and the mutual information declined with every level. The results of the performed ANOVA tests can be found as part of the Supplementary Materials.

## 5. Discussion

From the investigation and obtained results from the images used in this study, we can conclude that more details are obtained when a simpler wavelet is used for the wavelet-based decomposition of a grayscale medical image, contrary to the colored medical image. From the tested basic fusion rules, we conclude that the average fusion rule gives the best information, without a lack of or excess of information regarding the visual quality of the fused image. As also noted in a wavelet-based medical image fusion study [17], the maximum fusion rule (maximum selection rule) can amplify noise and artifacts due to their high contrast. This supports our conclusion that favors the average fusion rule out of the three fusion rules (average, minimum, and maximum).

Considering entropy as a quality metric and according to its higher values in all levels, ‘bior4.4’ emerges as the best wavelet for the wavelet-based image fusion among the wavelets evaluated in our experiments, and for the specific images in our experiments. We therefore find the highest entropy of the fused image to be achieved when the ‘bior4.4’ wavelet is selected. However, our results take into consideration only limited pairs of images from a single dataset and should not imply a general conclusion on the wavelet choice. It is interesting to note that researchers have conducted a comparative study [53] performing CT and PET image fusion with a gradient fusion rule using the ‘bior1.1’ and ‘bior4.4’ wavelets, achieving the highest entropy values with the ‘bior1.1’ among the other examined wavelets. This further supports the effectiveness of the biorthogonal wavelets in image fusion. It can be said that these findings are also in line with our results, again supporting the use of biorthogonal wavelets. The ‘bior4.4’ wavelet has also been used for multiple sclerosis detection, outperforming six other algorithms in sensitivity and accuracy [54], thus highlighting the potential usefulness of the ‘bior4.4’ wavelet for diagnosis purposes. Another recent study leverages the ‘bior4.4’ wavelet for obtaining sparse image representation in a compressive sensing approach to efficient and accurate image reconstruction [55].

Further, we have investigated the effect of the wavelet basis and fusion level on the values of the standard deviation and mutual information. Interestingly, the value for the standard deviation was highest when using the ‘bior1.3’ wavelet and the one-level maximum fusion rule. In other words, on the examined images, wavelets from the biorthogonal family achieve the highest values for two quality metrics (entropy (‘bior4.4’) and standard deviation (‘bior1.3’)). For both entropy and standard deviation, a single wavelet and fusion rule combination seems to achieve the highest values. We did not observe such consistency while examining the mutual information metric, as the results were more varied. Namely, ‘db1’ and ‘coif1’ wavelets achieved the highest values, but the fusion level and the fusion algorithm also varied.

Our study aimed to provide a detailed comparative evaluation of the impact that the selected wavelet has on the decomposition and medical image fusion, while at the same time exploring the potential of Python and PyWavelets. Although there are more complex fusion algorithms and schemes that can be found in existing literature, one of the points that makes our study stand out is the detailed comparative analysis it provides, one which can be further enriched with future studies. Segmentation offers a complementary route to supporting diagnosis and analysis of biomedical and medical images [56] and could be combined with medical image fusion. Advances in 6G-enabled telemedicine [57] are likewise expected to open new possibilities for novel artificial intelligence (AI)-based medical imaging pipelines.

## 6. Conclusions

The Python programming language fosters digital image processing research through available modules containing ready-to-use functions. The function for performing DWT within the PyWavelets package serves as a great example of how free and open-source software can help researchers explore the capabilities of wavelet-based medical image processing.

Nevertheless, there are several limitations of the present study. One limitation is the small variety of input medical images used for decomposition and fusion. The images are also part of the same dataset, and a more thorough assessment of the wavelet choice remains to be conducted as part of a future study on a broader scale. With the current study, we mostly aim to set a foundation for further, more elaborate studies, while focusing on reaffirming the potential of open-source software.

Furthermore, the lack of a ground-truth reference image for the fusion process only leaves room for assessment through parameters such as entropy, standard deviation, and mutual information. Consequently, some other parameters that require a comparison with a reference image have not been considered. Finally, the results obtained were not assessed by medical practitioners for practical value, accuracy, and relevance. Indeed, there is room for future research to confirm this study’s findings through both professional medical evaluation and comparison with non-wavelet-based fusion. Additionally, the influence of the wavelet family can be revisited and reassessed in more complex decomposition and fusion algorithms.

Future work could even take advantage of the rapid development in machine learning-, deep learning-, and artificial intelligence (AI)-based algorithms and further explore the potential values of different wavelet families and the DWT in supporting medical practice through diagnosis.

## Figures and Tables

**Table 1:** Normalized coefficient energy for the MRI image decomposition.

Wavelet	Normalized Coefficient Energy for the MRI Image Decomposition				
	Level	LL	LH	HL	HH
bior1.3	Level 1	0.9729	0.0099	0.0152	0.002
	Level 2	0.9421	0.0179	0.0309	0.0092
	Level 3	0.9213	0.0218	0.0444	0.0125
bior4.4	Level 1	0.9898	0.0038	0.0059	0.0005
	Level 2	0.9641	0.01	0.0188	0.0071
	Level 3	0.9548	0.0108	0.0255	0.0089
db1	Level 1	0.9745	0.0092	0.0142	0.002
	Level 2	0.9529	0.0144	0.0261	0.0067
	Level 3	0.9408	0.0176	0.0329	0.0087
db10	Level 1	0.989	0.0042	0.0062	0.0006
	Level 2	0.9592	0.0125	0.0203	0.008
	Level 3	0.9394	0.0153	0.032	0.0133
dmey	Level 1	0.9899	0.0042	0.0054	0.0005
	Level 2	0.9561	0.0152	0.0199	0.0088
	Level 3	0.934	0.0222	0.0289	0.015
coif1	Level 1	0.9833	0.0059	0.0096	0.0012
	Level 2	0.961	0.011	0.0213	0.0066
	Level 3	0.9473	0.0143	0.03	0.0084
coif10	Level 1	0.9895	0.0044	0.0056	0.0006
	Level 2	0.9508	0.015	0.0249	0.0092
	Level 3	0.9195	0.02	0.0438	0.0168

**Table 2:** Normalized coefficient energy for the PET image decomposition.

Wavelet	Normalized Coefficient Energy for the PET Image Decomposition				
	Level	LL	LH	HL	HH
bior1.3	Level 1	1	0	0	0
	Level 2	0.974	0.0099	0.0151	0.001
	Level 3	0.9312	0.024	0.0396	0.0052
bior4.4	Level 1	0.9927	0.0029	0.0042	0.0001
	Level 2	0.9955	0.0016	0.0027	0.0002
	Level 3	0.975	0.0088	0.014	0.0021
db1	Level 1	1	0	0	0
	Level 2	0.9753	0.0098	0.0139	0.0011
	Level 3	0.9435	0.0195	0.0326	0.0045
db10	Level 1	0.9927	0.003	0.0042	0.0001
	Level 2	0.9955	0.0019	0.0025	0.0002
	Level 3	0.9676	0.0113	0.0185	0.0026
dmey	Level 1	0.993	0.0028	0.0041	0.0001
	Level 2	0.9959	0.0018	0.0022	0.0002
	Level 3	0.9681	0.0109	0.0184	0.0025
coif1	Level 1	0.9924	0.003	0.0044	0.0001
	Level 2	0.9901	0.0041	0.0054	0.0003
	Level 3	0.9612	0.0137	0.0219	0.0032
coif10	Level 1	0.993	0.0028	0.0041	0.0001
	Level 2	0.9953	0.0022	0.0023	0.0002
	Level 3	0.9546	0.0147	0.0258	0.005

## Data Availability

The original contributions presented in this study are included in the article/supplementary material. Further inquiries can be directed to the corresponding authors.

## Supplementary Materials

The following supporting information can be downloaded at https://www.mdpi.com/article/10.3390/jimaging12080340/s1. Figure S1: Comparison of standard deviation values from fusion, using the average fusion rule: (a) S1aCTMRIAvgFusionRule (CT and MRI); (b) S1bPETGrayscaleMRIAvgFusionRule (PET grayscale and MRI); (c) S1cPETWithoutConversionMRIAvgFusionRule (PET without conversion and MRI); (d) S1dPETYUVMRIAvgFusionRule (PET YUV and MRI) (e) S1eFusionAVGRule (fusion results); using the minimum fusion rule: (f) S1fCTMRIMinFusionRule; (g) S1gPETGrayscaleMRIMinFusionRule; (h) S1hPETWithoutConversionMRIMinFusionRule; (i) S1iPETYUVMRIMinFusionRule; (j) S1jFusionMINRule; (k) S1kFusionMAXRule. Figure S2: Comparison of mutual information (MI) values from fusion using the minimum and maximum fusion rule. Figure S2a: minimum rule: (a) CT-Fused and MRI-Fused (CT and MRI fusion) and (b) PET-Fused and MRI-Fused (PET grayscale and MRI fusion). Figure S2b: minimum rule: (c) PET-Fused and MRI-Fused (PET without conversion and MRI fusion); (d) PET-Fused and MRI-Fused (PET YUV and MRI fusion). Figure S2c: maximum rule: (a) CT-Fused and MRI-Fused (CT and MRI fusion) and (b) PET-Fused and MRI-Fused (PET grayscale and MRI fusion). Figure S2d: maximum rule: (c) PET-Fused and MRI-Fused (PET without conversion and MRI fusion); (d) PET-Fused and MRI-Fused (PET YUV and MRI fusion). CSV files with ANOVA test results: anova_results_AVGFusPETWithoutConversionAndMRI.csv and anova_results_MAXFusPETWithoutConversionAndMRI.csv. The scripts used for decomposition, fusion, and quality metrics computations, as well as the ANOVA test script, can be found on the following GitHub link: https://github.com/Stojcher/python-medical-image-wavelet-decomposition-and-fusion/ (accessed on 30 April 2026).

## Abbreviations

The following abbreviations are used in this manuscript:

MRI	Magnetic resonance imagery
CT	Computed tomography
PET	Positron emission tomography
DWT	Discrete wavelet transform
IDWT	Inverse discrete wavelet transform
JPEG	Joint Photographic Experts Group
PSNR	Peak signal-to-noise ratio
SNR	Signal-to-noise Ratio
CNN	Convolutional neural network
IoT	Internet of things
EEG	Electroencephalography
AI	Artificial intelligence

## References

[1] Stojanov D., Koceski S. (2014). Topological MRI prostate segmentation method. Proceedings of the 2014 Federated Conference on Computer Science and Information Systems.

[2] Barbhuiya A.H.M.J.I., Hemachandran K. (2013). Wavelet Transformations & Its Major Applications In Digital Image Processing. Int. J. Eng. Res. Technol..

[3] Wang J., Huang K. (1996). Medical Image Compression by Using Three-Dimensional Wavelet Transformation. IEEE Trans. Med. Imaging.

[4] Kofidis E., Kolokotronis N., Vassilarakou A., Theodoridis S., Cavouras D. (1999). Wavelet-Based Medical Image Compression. Future Gener. Comput. Syst..

[5] Tackie Ammah P.N., Owusu E. (2019). Robust Medical Image Compression Based on Wavelet Transform and Vector Quantization. Inform. Med. Unlocked.

[6] Zerva M.C.H., Christou V., Giannakeas N., Tzallas A.T., Kondi L.P. (2023). An Improved Medical Image Compression Method Based on Wavelet Difference Reduction. IEEE Access.

[7] Ouahabi A. (2013). A Review of Wavelet Denoising in Medical Imaging. Proceedings of the 2013 8th International Workshop on Systems, Signal Processing and their Applications (WoSSPA).

[8] Pizurica A., Philips W., Lemahieu I., Acheroy M. (2003). A Versatile Wavelet Domain Noise Filtration Technique for Medical Imaging. IEEE Trans. Med. Imaging.

[9] Zangana H.M., Mustafa F.M. (2024). Hybrid Image Denoising Using Wavelet Transform and Deep Learning. EAI Endorsed Trans. AI Robot..

[10] Nitin, Gupta S.B. (2022). A Hybrid Image Denoising Method Based on Discrete Wavelet Transformation with Pre-Gaussian Filtering. Indian J. Sci. Technol..

[11] Zhu G., Wan M., Cao W., Yu Z., Hu J., Li B., Fan X. (2026). Med-D3CG: Wavelet-Based Diffusion in the Difference Domain for Cross-Modality Medical Image Generation. Expert Syst. Appl..

[12] AlZubi S., Islam N., Abbod M. (2011). Multiresolution Analysis Using Wavelet, Ridgelet, and Curvelet Transforms for Medical Image Segmentation. Int. J. Biomed. Imaging.

[13] Li H.-A., Fan J., Yu K., Qi X., Wen Z., Hua Q., Zhang M., Zheng Q. (2020). Medical Image Coloring Based on Gabor Filtering for Internet of Medical Things. IEEE Access.

[14] Sui K., Kim H.-G. (2019). Research on Application of Multimedia Image Processing Technology Based on Wavelet Transform. EURASIP J. Image Video Process..

[15] Ali A., Dutta A., Kaplun D., Romanov S., Sarkar R. (2026). A Lightweight Wavelet Group Equivariant Convolution Neural Network for Medical Image Classification. Int. J. Comput. Intell. Syst..

[16] Khanbabaei H., Mohammadi S., Sadoughi H.-R. (2025). Applications of Wavelet Transforms in Radiomic Feature Extraction from Medical Images: A Systematic Review. J. Res. Health.

[17] Yang Y., Park D.S., Huang S., Rao N. (2010). Medical Image Fusion via an Effective Wavelet-Based Approach. EURASIP J. Adv. Signal Process..

[18] Alzahrani A.A. (2024). Enhanced Multimodal Medical Image Fusion via Modified DWT with Arithmetic Optimization Algorithm. Sci. Rep..

[19] Bhavana V., Krishnappa H.K. (2015). Multi-Modality Medical Image Fusion Using Discrete Wavelet Transform. Procedia Comput. Sci..

[20] Rani K., Sharma R. (2013). Study of Different Image Fusion Algorithm. Int. J. Emerg. Technol. Adv. Eng. IJETAE.

[21] Anusha K., Pragnesh P., Giridhar T., Ajay R. (2025). Wavelet Transform Based Image Fusion for Brain Tumour Detection. J. Electron. Autom. Eng..

[22] Bhosekar S., Singh P., Garg D., Ravi V., Diwakar M. (2025). A Review of Deep Learning-Based Multi-Modal Medical Image Fusion. Open Bioinforma. J..

[23] Pal B., Jain S. (2022). Novel Discrete Component Wavelet Transform for Detection of Cerebrovascular Diseases. Sādhanā.

[24] Jain N., Yadav A., Kumar Sariya Y., Balodi A. (2021). Analysis of Discrete Wavelet Transforms Variants for the Fusion of CT and MRI Images. Open Biomed. Eng. J..

[25] Zubair M., Hussain M., Albashrawi M.A., Bendechache M., Owais M. (2025). A Comprehensive Review of Techniques, Algorithms, Advancements, Challenges, and Clinical Applications of Multi-Modal Medical Image Fusion for Improved Diagnosis. Comput. Methods Programs Biomed..

[26] Ravi J., Narmadha R. (2024). Optimized Dual-Tree Complex Wavelet Transform Aided Multimodal Image Fusion with Adaptive Weighted Average Fusion Strategy. Sci. Rep..

[27] Tian C., Zhang J., Tang L. (2025). Perceptual Objective Evaluation for Multimodal Medical Image Fusion. Front. Phys..

[28] Wang Y., Zhang R., Zhang Z., Liu N., Wang X. (2026). 3DWaFusion: Three-Dimensional Multiscale Wavelet Convolutional Neural Network for Multimodal Medical Image Fusion. Sensors.

[29] Sreelakshmi A.N., Sujatha N. (2026). A Novel Medical Image Fusion Approach Using Local Features and Deep Learning in Wavelet Domain. Int. J. Artif. Intell. Mach. Learn..

[30] Hayath T.M., Sai Madhavi D. (2026). Medical Image Fusion Using Symmetric and Asymmetric Strategies: Evaluating Classical and Deep Learning Methods. Eng. Technol. Appl. Sci. Res..

[31] El-Shafai W., Ghandour C., El-Rabaie S. (2026). Advancements in Medical Image Fusion: A Comprehensive Study on RTVD Framework and FPGA Deployment Challenges. Multimed. Tools Appl..

[32] Preethika S., Bharath S., Navaneethan S., Arunkumar K. Performance Analysis of MCSD and Wavelet-Based Fusion for Multimodal Medical Imaging. Proceedings of the 2026 International Conference on Smart Futuristic Technology (ICSFT).

[33] Wang W., Yan D., Wu X., He W., Chen Z., Yuan X., Li L. (2023). Low-Light Image Enhancement Based on Virtual Exposure. Signal Process. Image Commun..

[34] Shakibania H., Raoufi S., Khotanlou H. (2025). CDAN: Convolutional Dense Attention-Guided Network for Low-Light Image Enhancement. Digit. Signal Process..

[35] Sethi K., Sahoo B., Shrishti, Pavan Kumar B.N., Dhal K., Cho W., Joshi G.P. (2025). Lightweight DWT Steganography With ECC-ChaCha20 for Secure Medical IoT Systems. IEEE Access.

[36] Guo T., Zhang T., Lim E., Lopez-Benitez M., Ma F., Yu L. (2022). A Review of Wavelet Analysis and Its Applications: Challenges and Opportunities. IEEE Access.

[37] Mishra H.O.S., Bhatnagar S. (2014). MRI and CT Image Fusion Based on Wavelet Transform. Int. J. Inf. Comput. Technol..

[38] Daubechies I. (1992). Ten Lectures on Wavelets.

[39] Amolins K., Zhang Y., Dare P. (2007). Wavelet Based Image Fusion Techniques—An Introduction, Review and Comparison. ISPRS J. Photogramm. Remote Sens..

[40] Misiti M., Misiti Y., Oppenheim G., Poggi J.-M. (2004). Wavelet Toolbox User’s Guide.

[41] Jagalingam P., Hegde A.V. (2015). A Review of Quality Metrics for Fused Image. Aquat. Procedia.

[42] Azam M.A., Khan K.B., Salahuddin S., Rehman E., Khan S.A., Khan M.A., Kadry S., Gandomi A.H. (2022). A Review on Multimodal Medical Image Fusion: Compendious Analysis of Medical Modalities, Multimodal Databases, Fusion Techniques and Quality Metrics. Comput. Biol. Med..

[43] Havard Medical Image Fusion Datasets. GitHub Repository. https://github.com/xianming-gu/Havard-Medical-Image-Fusion-Datasets?tab=readme-ov-file.

[44] Pillow Developers (2024). *PIL.Image.Image.convert()*; Pillow Version 10.2.0, Pillow Documentation. https://pillow.readthedocs.io/en/stable/reference/Image.html.

[45] Li Z.-N., Drew M.S. (2004). Fundamentals of Multimedia.

[46] Van Rossum G., Python Development Team (2018). Python Tutorial.

[47] Anderson E.W., Preston G.A., Silva C.T. (2010). Using Python for Signal Processing and Visualization. Comput. Sci. Eng..

[48] Lee G., Gommers R., Waselewski F., Wohlfahrt K., O’Leary A. (2019). PyWavelets: A Python Package for Wavelet Analysis. J. Open Source Softw..

[49] Ali T.J., Akhtar P., Faris M., Mala I., Zia S.S. (2016). Implementation of Discrete Fourier Transform And Orthogonal Discrete Wavelet Transform In Python. J. Indep. Stud. Res. Comput..

[50] Applied & Computational Mathematics Lab 8: Introduction to Wavelets 2019. https://acme.byu.edu/00000179-afb2-d74f-a3ff-bfbb158e0000/wavelets19-pdf.

[51] Chen C.-I., Lan C.-K., Chen Y.-C., Chen C.-H., Chang Y.-R. (2020). Wavelet Energy Fuzzy Neural Network-Based Fault Protection System for Microgrid. Energies.

[52] Abhijith S. (2017). Wavelet Bayes Adaptive Image Denoising. Int. Res. J. Adv. Eng. Sci..

[53] Indhumathi R., Kurunathan H., Kumar U.S., Marshiana D., Indira K.P. (2024). Multi-Resolution Bi-Orthogonal Wavelet Transforms Using Gradient Fusion Rule for Multimodal Medical Image Fusion. Afr. J. Biomed. Res..

[54] Wang S.-H., Zhan T.-M., Chen Y., Zhang Y., Yang M., Lu H.-M., Wang H.-N., Liu B., Phillips P. (2016). Multiple Sclerosis Detection Based on Biorthogonal Wavelet Transform, RBF Kernel Principal Component Analysis, and Logistic Regression. IEEE Access.

[55] Rakotonirina H., Luc S., Randrianandrasana M. (2025). Image Reconstruction in Compressive Sensing Using the Level 3 Biorthogonal 4.4 (Bior4.4) Discrete Wavelet Transform and SP, CoSaMP and ALISTA Algorithm. Am. J. Electr. Comput. Eng..

[56] Vetova S., Lazarova M. Biomedical Image Processing Workflow Using Segmentation. Proceedings of the 2023 International Scientific Conference on Computer Science (COMSCI).

[57] Sotirova E., Poulkov V., Manolova A., Sotirov S. 6G-Enabled Telemedicine and AI-Based Medical Imaging: A Hybrid Modular Neural Network with Intuitionistic Fuzzy Logic for Atypical Pneumonias in Childhood. Proceedings of the 2026 Joint International Conference on Digital Arts, Media and Technology with ECTI Northern Section Conference on Electrical, Electronics, Computer and Telecommunication Engineering (ECTI DAMT & NCON).

